# Beta 2 Adrenergic Receptor Antagonist Propranolol and Opioidergic Receptor Antagonist Naltrexone Produce Synergistic Effects on Breast Cancer Growth Prevention by Acting on Cancer Cells and Immune Environment in a Preclinical Model of Breast Cancer

**DOI:** 10.3390/cancers13194858

**Published:** 2021-09-28

**Authors:** Sengottuvelan Murugan, Bénédicte Rousseau, Dipak K. Sarkar

**Affiliations:** Endocrine Research Program, Department of Animal Sciences, Rutgers, The State University of New Jersey, 67 Poultry Farm Lane, New Brunswick, NJ 08901, USA; s.murugan@rutgers.edu (S.M.); br375@scarletmail.rutgers.edu (B.R.)

**Keywords:** beta-adrenergic blocker, mu-opioid receptor antagonist, breast cancer cells, innate immune system, cell growth arrest, apoptosis, epithelial–mesenchymal transition factors, tumor xenograft

## Abstract

**Simple Summary:**

Here, we show that propranolol, a beta blocker used to treat high blood pressure, and naltrexone, an opiate antagonist used for drug and alcohol dependence, when combined, produce marked inhibitory effects on tumor growth and tumor mass while improving the survival rate, increasing NK cell activity, and reducing inflammatory cytokine levels in plasma. These antitumor effects resulted from a reduction in tumor cell proliferation, the induction of cellular apoptosis, and the prevention of epithelial–mesenchymal transition in the tumor. Our data identify a novel treatment with a combination of approved classes of drugs in preclinical breast cancer models.

**Abstract:**

Cancer progression is known to be promoted by increased body stress caused by elevated beta-adrenergic and opioidergic nervous system activities. The effects of β2-adrenergic blocker propranolol (PRO) and μ-opioid receptor antagonist naltrexone (NTX) were tested using a preclinical model of human breast cancer. These drugs, individually, and more potently when combined, inhibited the cell growth and progression of breast cancer cells in vitro in cultures, and in vivo in rat xenografts. The antitumor activities of these drugs were associated with direct cell intrinsic effects, including increased cell growth arrest, elevated levels of apoptotic proteins, and reduced production of epithelial–mesenchymal transition factors by the tumor cells, as well as effects on innate immune activation and reduced inflammatory cytokine levels in plasma. These data suggest that the combined treatments of PRO and NTX produce impressive antitumor effects in the preclinical breast cancer model, and thereby may provide a new combinatorial treatment strategy with more clinical treatment modalities.

## 1. Introduction

Breast cancer is the second most prevalent cancer after lung cancer among American women. The National Cancer Institute estimated that there would be 41,760 deaths due to breast cancer and 268,300 new cases of breast cancer among American women for the year 2019 [1]. Breast tumors vary in their molecular subtypes: luminal A, luminal B, triple negative/basal-like, and Her2 type. The prevalence rates of the four subtypes of breast cancer appear to differ by race. For example, the triple negative/basal type, a type which has a poor prognosis, is more common among younger black women, while the luminal A tumor type, which has the best prognosis of the subtypes, occurs less often among black women than white women [2,3]. Immune response is considered to be an important prognostic factor in the tumor microenvironment of both HER2^+^ and basal tumors [4].

Clinical studies have suggested that stress, chronic depression, and social support might influence cancer onset and progression [5]. Stress can also affect many aspects of the body’s immune systems. For example, higher levels of stress were shown to be associated with a decrease in natural killer (NK) cell lysis activity, macrophage migration activity, T cell population, lymphocyte proliferation following infection, and interferon-γ (IFN-γ) levels [6]. These factors are reported to be important components of immunity against cancer [7,8]. Therefore, controlling the body’s stress response may be beneficial to increasing immunity and fighting against cancer. Epinephrine and norepinephrine are classic neurotransmitters that mediate stress responses from the autonomic nervous system. These catecholamines bind to adrenergic receptors, which belong to the G protein coupled receptors (GPCRs). One member of the group of adrenergic receptors is β2-AR (B2AR), which has been implied in different processes of breast cancer initiation and progression [9]. B2ARs have been identified in human breast cancer and breast cancer cell lines MDA-MB-231, MCF-7, VHB-1, T47-D, and BT-20 [10]. In vivo studies of B2AR signaling in animal models of breast tumors have demonstrated an association with increased nodal involvement and the development of tumor metastasis [11]. It has also been shown that these effects are inhibited by the nonselective B2AR antagonist propranolol (PRO) [12]. Recently, a series of matched population-based observational studies were performed to determine associations between the use of PRO and the risk of local tumor invasion, nodal and metastatic involvement at diagnosis, and breast cancer-specific mortality. This study demonstrated that reducing the B2AR signaling pathway is beneficial to suppressing breast cancer progression and mortality. PRO has also been suggested to have strong anti-metastatic effects in breast cancer [13]. Opioid receptors also participate in the regulation of mood and stress responsivity [14]. Cortisol increases are a core component of the response to psychological stress, and cortisol in turn serves to regulate the systemic components of the stress response, therefore contributing to long-term homeostasis [15]. Cortisol secretion and its feedback effects are intimately tied to the endogenous opioid system, particularly the µ-opioid receptor (MOR) system [16]. A single-nucleotide polymorphism (SNP) of MOR within the OPRM1 gene, A118G, has been implicated in drug addiction, pain sensitivity, and stress response, as well as in the treatment response to the µ-opioid antagonist naltrexone (NTX) [17]. Interestingly, a strong association between G allele presence at MOR A118G and increased breast cancer incidence in females has been reported and is interpreted as a highly significant risk factor in breast cancer development [18]. In vitro research suggests that treatment with MOR antagonists inhibits cancer proliferation and shows some promise for attenuating tumor growth in humans, thereby enhancing survival [19]. A low dose of NTX has been shown to have promising results for people with primary cancer of various tissues, including breast tissue [20].

Opioid receptors and beta-adrenergic receptors have been shown to coexist in many cells, including immune cells and breast tumor cells, and have been shown to act via GPCRs, which are known to heterodimerize with closely related members, resulting in the modulation of their functions [21]. Ligand binding studies show that opioidergic and adrenergic agents can bind to their complementary receptors and influence the activity of other compound types through an allosteric mechanism [21], which supports the notion that a combination of opioidergic and adrenergic agents could have complementarity cellular processes. Despite this evidence, the complementary physiological activities of combined B2AR and OR agents in regulating immune cell functions and tumor growth have not been determined. Therefore, in this study, we investigate if combination treatments of B2AR antagonist PRO and MOR antagonist NTX will decrease the tumor growth of breast cancers in a synergistic manner. We provide evidence that this combination treatment of PRO and NTX has potent anticancer effects, and we show the possible clinical applicability.

## 2. Materials and Methods

Information about Research Resource Identifier (RRID) for each relevant reagent is provided in Tables S1–S3.

### 2.1. Breast Cancer Cell Cultures

Human breast cancer cells MDA-MB-231 (RRID:CVCL_0062), MDA-MB-468 (RRID:CVCL_0419), and T47D (RRID:CVCL_0553) were obtained from American Type Culture Collection (ATCC; Rockville, MD, USA), and MDA-MB-231/Luc-GFP cells were purchased from GenTarget Inc. (San Diego, CA, USA; RRID-N/A). These cells were maintained in cultures with the medium suggested by the supplier at 37 °C in a humid environment containing 5% CO_2_. Using STR profile authenticated cell lines, we first confirmed that these human breast cancer cells maintained their morphological phenotype and expressed primary marker receptors (ER, PR, and HER2) during passages as described elsewhere [22].

### 2.2. Immunoprecipitation and Immunoblotting Analysis of Beta-Adrenergic and Opioid Receptors

For immunoprecipitation we used the Pierce Classic IP Kit (Thermo Scientific, Branchburg, NJ, USA). One milligram of cell lysate was precleared using the Control Agarose Resin following the methods described previously [23]. Lysates were then solubilized in lysis buffer before incubation with anti-B2AR (RRID:AB_940556) antibody overnight at 4 °C. Following the incubation, the antibody/lysate complex was captured with 20 µL of Pierce Protein A/G Agarose for 1 h. After elution using the sample buffer, the immune complex was analyzed by Western blot analyses using antibodies against MOR (RRID:AB_177512) and β-actin (RRID:AB_2242334; used as a normalizing protein). Experiments were performed in triplicate.

### 2.3. Cell Viability, Colony Formation, Migration, and Invasion Assays

Cells were plated in 96-well plates (2000 cells/well), allowed to adhere overnight, and treated with PRO and NTX alone or in combination with increasing concentrations (0.001 µM to 200 µM) for 24, 48, and 72 h in quadruplicate wells, and at the end of the drug treatment period, run through MTT assay for determination of cell viability. The values of the inhibitory concentration required to arrest 50% of the cells (IC50) were calculated for individual or drug combinations. To determine whether the NTX + PRO combination (1:1 ratio) has synergistic, additive, or antagonistic activity on cell lines, we used isobologram and combination index methods, as per the principle of Chou and Talalay [24] using the CalcuSyn software (Biosoft, Ferguson, MO, USA). Data obtained from the growth inhibitory experiments (MTT assay) were used to perform these analyses. The isobologram method is a graphical representation of the pharmacologic interaction and is formed by selecting a desired fractional cell kill (Fa) and plotting the individual drug doses required to generate that Fa on their respective x- and y-axes. A straight line is then drawn to connect the points. The observed dose combination of the two agents that achieved that particular Fa is then plotted on the isobologram. The combination index (CI) method is a mathematical and quantitative representation of a two-drug pharmacologic interaction. Using data from the growth inhibitory experiments and computerized software, CI values are generated over a range of Fa levels from 0.05 to 0.90 (5–90% growth inhibition). If the CI values are <1, it means the drugs have a synergistic effect. If CI values are equal to 1, it means the drugs have additive effect, or if CI values are >1 it means the drugs have antagonistic activity.

For clonogenic, cell migration, and cell invasion assays, a drug concentration of 100 μM was chosen for individual or drug combinations. The clonogenic assay was performed as previously described [25]. Cell migration assay was performed following the procedure described previously [26]. In brief, the upper insert chambers (8-μm pore polycarbonate membrane) were seeded with cells at a density of 5 × 10^4^ cells (cell numbers established by preliminary experiments) per well in the appropriate serum-free medium. A 10% FBS-containing medium was then added to the lower chamber to serve as a chemoattractant. A 100 μM concentration of beta-adrenergic and opioidergic drugs alone or in combination or vehicle alone was added to both lower and upper chambers, and cells were allowed to migrate across the membrane for 12 h, at which point the cells were fixed in 100% methanol for 30 min and stained with 0.5% crystal violet for calculating the number of migrated cells in each group. Cell invasion assay was performed by transmigration through an extracellular matrix (ECM) by a previously described method [26]. In brief, the upper chamber of the matrix in 24-well Matrigel invasion chamber plates (BD BioCoat™ Matrigel™ Chamber, BD Biosciences, San Jose, CA, USA) was loaded with 5 × 10^4^ cells in 500 μL of the appropriate medium (cell numbers established by preliminary experiments). After 24 h, medium containing 1% FBS was used on both the lower and upper surfaces. One day later, the medium of the upper chamber was replaced with 500 μL of medium containing 1% FBS without any drug treatment. The medium was replaced with medium containing 10% FBS and 25 ng/mL EGF in the lower chamber. The effect of beta-adrenergic and opioidergic drugs was tested by adding a 100 μM concentration of these drugs alone or in combination or vehicle alone in both lower and upper chambers. After 48 h of incubation, the noninvasive cells that remained within the inserts were removed with a cotton swab. Cells that traversed through the Matrigel and the polycarbonate filter (8-μm pore size) attached to the lower surface were stained with 0.5% crystal violet for calculating the invasion index.

### 2.4. Cell Cycle Analysis

Cell cycle analysis of breast cancer cells was carried out using propidium iodide (PI) staining protocol according to the manufacturer’s instructions (Nexcelom Bioscience LLC, Lawrence, MA, USA). Briefly, breast cancer cells were seeded at a density of 1 × 10^6^ in 10 cm^2^ culture dishes and grown to 50% confluence and were treated with a 100 μM concentration of PRO or NTX alone or in combination or vehicle alone for 48 h. Differences in fluorescence intensity were used to determine the percentage of cells in each phase of the cell cycle and represented as histograms.

### 2.5. Animal Maintenance

T-cell-deficient, athymic nude (Crl: NIH-Foxn1rnu) female rats aged 21–28 days old were purchased from Charles River (Portage, MI, USA) and maintained in a pathogen-free condition with a 12-h light/dark cycle at our institute’s animal research facility. Animal care and treatment were performed in accordance with institutional guidelines, and protocols were approved by the Rutgers Institutional Animal Care and Facilities Committee and complied with National Institutes of Health policy.

### 2.6. Subcutaneous Xenograft Experiments

MDA-MB 231 cells, at a final concentration of 1 × 10^7^ cells/rat in 200-µL of PBS-50% Matrigel (BD Biosciences, San Jose, CA, USA) mixture, were injected subcutaneously (SC) into the right flank of the athymic nude female rats (Crl: NIH-Foxn1rnu; Charles River; Portage, MI, USA). After tumors reached a diameter of approximately 50 mm^3^, the animals were randomly assigned to different treatment groups and injected s.c. daily with saline (control), NTX (10 mg/kg), PRO (10 mg/kg), or a combination of NTX and PRO for 4 weeks. Tumor shrinkage/growth was measured in animals daily and animal weights were measured every other day. Animals were euthanized when the tumor reached 5000 mm^3^. Three dimensions of the tumor were measured using electronic calipers, and tumor volumes were calculated by the formula L × W2/2. The mean ± SEM of tumor volume was calculated weekly for each experimental group and presented. After the animals were sacrificed, the tumors were excised, weighed, sized, and photographed to visualize differences in tumor morphology. A portion of the excised tumors was snap-frozen by immersion in liquid nitrogen and stored at −80 °C until further use.

For the survival study, the tumor-bearing animals were monitored daily until the animals demonstrated an obvious health deterioration, or the maximum tumor volume allowed by ethical standards was reached; therefore, data are not truly absolute for animal survival. At the endpoint (prior to the onset of death), the animals were euthanized and the survival curves were plotted in a Kaplan–Meier survival curve.

For the bioluminescent imaging study, the animals were injected with MDA-MB-231/Luc-GFP cells (GenTarget Inc., San Diego, CA, USA; RRID-N/A) and followed the same drug treatment regimens for 6 weeks. Under isoflurane anesthesia, bioluminescent signals at the injection site (right flank) were detected by injecting i.p. with D-luciferin dissolved in 1× PBS at a concentration of 60 mg/kg (PerkinElmer Health Sciences Inc., Shelton, CT, USA) and transferring the animals to a light-tight chamber for optical imaging (In Vivo FX PROO, Bruker Corp., Billerica, MA, USA) using a charge-coupled device (CCD) camera (2048 × 2048 pixels) over a period of 5 min. The signal intensity (SI) is expressed as average photon flux (photo/sec) from the tumor.

### 2.7. Histopathology and Immunohistochemistry

At the end of the experiment, a portion of the tumor tissues was fixed in 10% neutral buffered formalin overnight and embedded in paraffin, was cut into 5-μm-thick sections, and some of these tissue sections were stained with H&E and a histopathological evaluation of tumors (mitotic cells) was conducted as previously described [27]. The rest of the sections were stained for various proteins using the ABC Elite Vectastain Kit (Vector Labs) according to the manufacturer’s instructions using various primary antibodies. Details of all primary antibodies used are described in Table S1.

### 2.8. Immunoblotting Detection of Proteins from Immune Cells

For protein analyses, tumor tissues from in vivo experiments, enriched NK cells from the spleen, and NK-92 MI cancer cells were lysed with a buffer containing protease and phosphatase inhibitors (25 mM Tris-HCl, pH 7.4; 150 mM NaCl; 1% Nonidet P-40; 1 mM EDTA; and 5% glycerol with Pierce Halt Protease Inhibitor), and 50 mg of protein from total lysate was used for measurements of various proteins using various antibodies (Table S1) and Western blot analysis [27]. Each protein was normalized to corresponding intensities for β-actin.

### 2.9. Enrichment of NK Cells from Spleen and PBMCs and the NK Cell Cytolytic Assay

The spleen tissues from control and drug-treated rats were processed, and RBCs and granulocytes were removed from splenocyte suspensions by density centrifugation using Histopaque 1083 (Sigma-Aldrich, St. Louis, MO, USA) as previously described [27]. PBMCs from rats were isolated from freshly obtained heparinized whole blood using the SepMate 15 mL tube (Stemcell Technologies, Vancouver, BC, USA) according to the manufacturer’s instructions. Enrichment of NK cells from splenocytes and PBMCs was performed by antibody-attached microbeads according to the manufacturer’s instructions (Miltenyi Biotec, Somerville, MA, USA). The cytotoxic function of these NK cells was evaluated by calcein-acetoxymethyl (AM) ester release assay using NK cell sensitive YAC-1 cells (ATCC; Cat# TIB-160, Rockville, MD; RRID:CVCL_2244) as described previously [27].

### 2.10. Flow Cytometry Analysis of Immune Cells

PBMC samples and splenocytes were prepared as described above. Tumor tissue was minced with a scalpel blade using a crisscross method and ground with 1 mL of ACK lysing buffer to remove red blood cells. The cell suspensions were filtered through a 70-micron sieve and then washed with PBS. Cells from the tumor were collected and dispersed with 100 μL of PBS and stained by the addition of fluorescence-conjugated antibodies. A list of antibodies, dye, isotype controls, and beads used for the flow cytometry experiment is given in Tables S2 and S3. Immune cells from the tumor, PBMCs, and splenocyte samples were examined by following the gating strategy as described previously [26]. The data collected from each sample were exported and analyzed using FlowJoTM version 10.7.

### 2.11. Cytokine Multiplex Immunoassay

After the end of the drug treatment period, plasma samples from control and drug-treated rats were processed and used for cytokine detection using the ProcartaPlex multiplex immunoassay using Luminex^®^ xMAP^®^ (multianalyte profiling) technology and the protocols described by the manufacturer and analyzed with the LuminexTM instrument (MAGPIX^®^ xPONENT^®^, Life Technologies Corporation, Grand Island, New York, NY, USA). Median fluorescence index (MFI) is calculated and presented.

### 2.12. Statistical Analysis

Statistical analysis was performed using GraphPad Prism 5. The significance between treatment and controls was assessed using one-way ANOVA between treatments and two-way ANOVA between treatments and days of exposure. For survival data, Kaplan–Meier curves were generated using Graph Pad Prism. *p* < 0.05 was considered significant.

## 3. Results

### 3.1. Verification That Breast Cancer Cells Contain Beta-Adrenergic and Opioidergic Receptors

**Figure 1:**
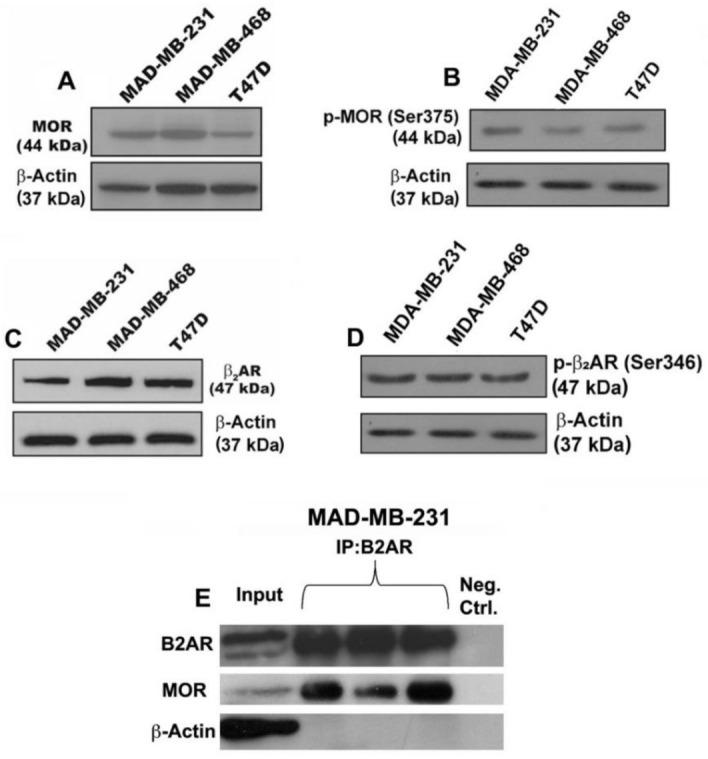
Breast cancer cells express μ-opioid receptor (MOR) and β2-adrenergic receptor (B2AR) and their dimers. Upper panel pictures show immunoblotting data of MOR (**A**) and phospho-MOR (**B**) in human breast cancer cells MDA-MB-231, MDA-MB-468, and T47D. Middle panel pictures are B2AR (**C**) and phospho-B2AR (**D**) in these cells. Lower panel pictures are immunoblotting data of B2AR or MOR after performing immunoprecipitation (IP) with B2AR on MDA-MB-231 cells (**E**). Input is the control immunoblotting of protein lysate and IP results are shown in triplicate. β2-actin is used as a house-keeping control protein.

We determined the effects of NTX and PRO alone or in combination on the growth and proliferation of three human breast cancer cells: MDA-MB-231, MDA-MB-468, and T47D. We first verified that these three cell lines expressed MOR (Figure 1A), phospho-MOR (Figure 1B), B2AR receptors (Figure 1C), and phospho-B2AR (Figure 1D) by immunoblotting. We also identified that MOR and B2AR can physically interact in MDA-MB-231 cells as determined by immunoprecipitation with B2AR antibody and probing with B2AR or MOR antibodies (Figure 1E).

### 3.2. Effects of Beta-Adrenergic and Opioidergic Drugs Alone or in Combination on Cancer Cell Viability, Clonogenicity, Migration, and Invasion

**Figure 2:**
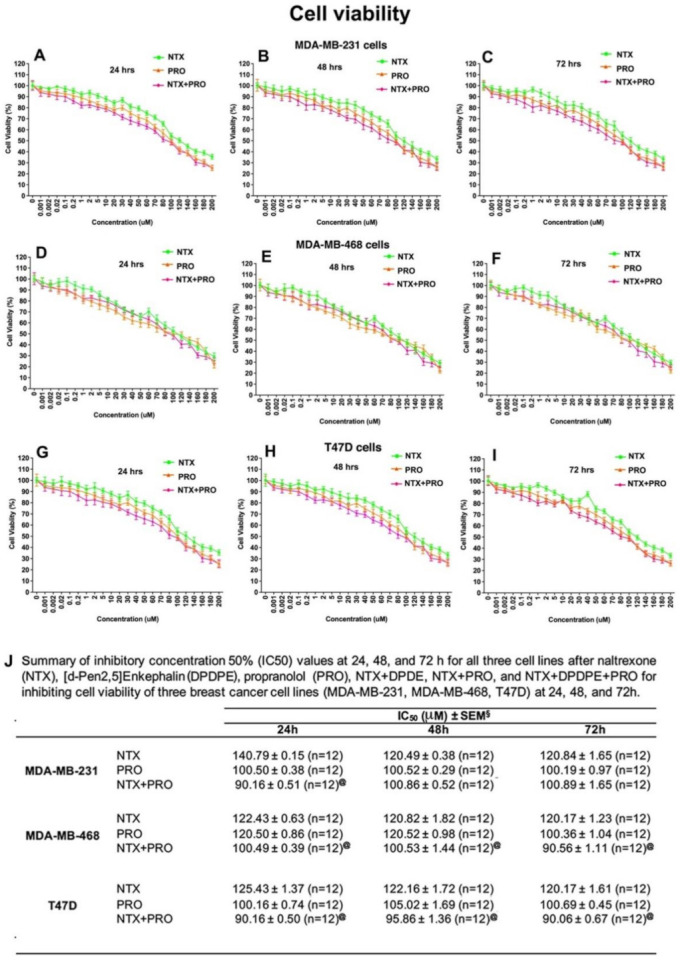
Propranolol (PRO) and naltrexone (NTX) produce synergistic effects on reduction in breast tumor cell viability. Cells were cultured in the presence of the indicated doses of a single drug (NTX or PRO) or drug combination (NTX + PRO) on viability of MDA-MB-231 cells (**A**–**C**), MDA-MB-468 cells (**D**–**F**), and T47D cells (**G**–**I**) after 24, 48, and 72 h. Cell viability was analyzed by MTT assay and data presented as mean ± SEM (*n* = 12). The values of the inhibitory concentration required to arrest 50% of the cells (IC50) were calculated and presented in (**J**).

The direct effect of opioidergic and adrenergic agents on breast cancer cell viability was tested following treatment with various concentrations (0.001 µM to 200 µM) of NTX and PRO alone or in combination on MDA-MB-231, MDA-MB-468, and T47D cells for 24, 48, and 72 h. These drugs, either individually or in combination, caused a dose-dependent inhibition of cell viability at all time points (Figure 2A–I). The IC50 dose varied between different treatment groups and times of treatment (Figure 2J). The combination of the two drugs displayed the most potent growth inhibition with the IC50 of ∼ 90–100 µM in all cell lines and treatment times. To determine whether the NTX + PRO combination (1:1 ratio) has synergistic, additive, or antagonistic activity on the three cell lines at different time points, we calculated combination index (CI) values using CompuSyn software and IC50 values at 24, 48, and 72 h for the three cell lines. Based on the CI values, it is clear that the IC50 dose of the NTX + PRO combination has a synergistic effect on the three cell lines at all time points studied (Table S4).

**Figure 3:**
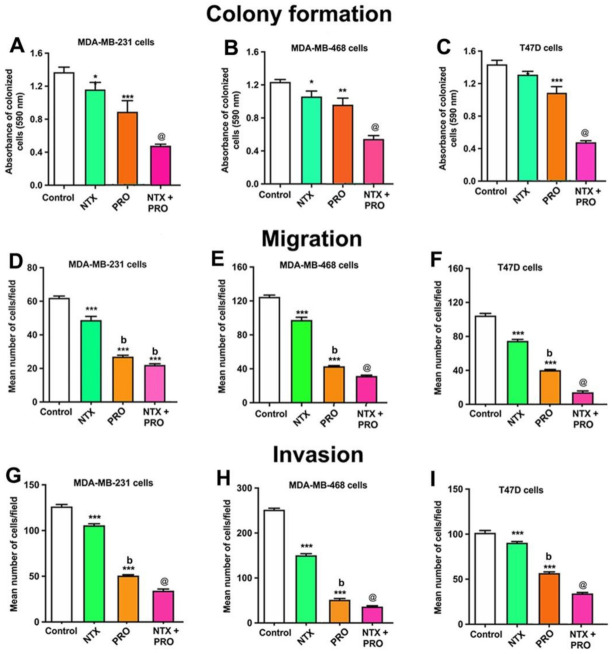
Propranolol (PRO) and naltrexone (NTX) reduce clonogenic behavior (**A**–**C**), cell migration (**D**–**F**), and cell invasion (**G**–**I**) in MDA-MB-231 cells, MDA-MB-468 cells, and T47D cells. (**A**–**C**). Clonogenic behavior of MB-231 cells, MDA-MB-468 cells, and T47D cells was determined after 14 days of treatment with a 100 μM dose of PRO and NTX, alone or in combination. The optical density (OD) values of staining are shown in histograms. (**D**–**F**). The effects of PRO and NTX alone or in combination for a period of 12 h on cell migration (number of cells/field) are shown as histograms for MDA-MB-231 (**D**), MDA-MB-468 (**E**), and T47D (**F**) cells. (**G**–**I**). Effects on cell invasion after 48 h of treatment with these drugs. Histograms depict the number of cells invaded/field for MDA-MB-231 (**G**), MDA-MB-468 (**H**), and T47D (**I**) cells. Data are mean ± SEM values obtained from four independent experiments in duplicate. Statistical significance was determined using one-way ANOVA with the Newman–Keuls post hoc test. *, *p* < 0.05, **, *p* < 0.01, ***, *p* < 0.001 compared to control, b, *p* < 0.05, compared with NTX, @, *p* < 0.05, compared with the rest of the groups.

Because the IC50 dose of these agents ranged between 90 and 100 µM in the cell viability assay, we used a 100 µM dose for all agents for clonogenicity assays. In all three cells, NTX and PRO alone showed moderate inhibition; NTX in combination with PRO showed maximal inhibition (Figure 3A–C and Figure S1A–C).

The effects of the 100 µM dose of beta-adrenergic and opioidergic agents on the cell mobility of three cell lines are shown in Figure 3D–F and Figure S1D–F. These data indicate that single drugs, NTX or PRO alone, produced significant but moderate inhibitory effects on all cell lines. A combination of NTX + PRO produced significantly more inhibition of cell migration than each drug alone in these three cell lines.

The effectiveness of a 100 µM dose of these two agents on the cell invasion of all cell lines is shown in Figure 2G–I and Figure S1G–I. The results show that NTX and PRO alone and in combination decreased cell invasion in these three cell lines.

### 3.3. Effects on Cell Cycle Changes in Cancer Cells

**Figure 4:**
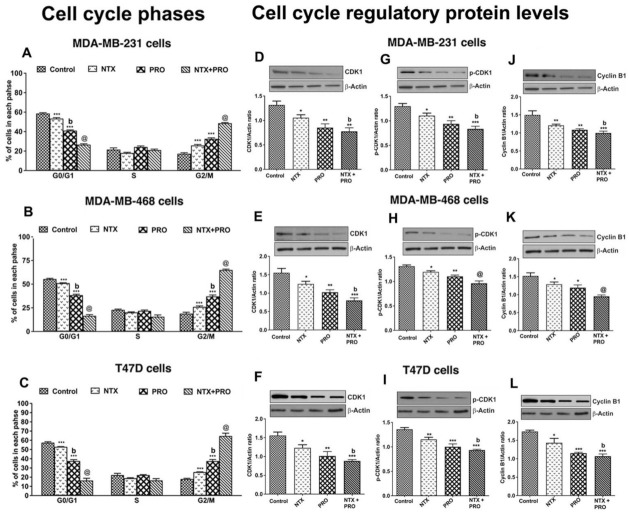
PRO and NTX increase G2/M arrest (**A**–**C**) and modulate levels of G2M-regulatory proteins (**D**–**L**) in MDA-MB-231, MDA-MB-468, and T47D cells. (**A**–**C**). Cells were treated with a 100 μM concentration of the drugs alone or in combination or vehicle alone for 48 h. After the treatment period, cells were stained with PI and analyzed for cell cycle distribution using flow cytometry. Proportions of cells in each phase were quantified and the values are shown as histograms. (**D**–**L**). After the drug treatments, some cells were extracted and used for Western blot analysis of CDK, phospho-CDK, and Cycline B1. Representative blots are presented on the top and mean densitometric values are presented as ratio of β-actin in the histograms. Data presented are mean ± SEM (*n* = 5–6 samples/group) and were analyzed using one-way ANOVA with the Newman–Keuls multiple comparisons post hoc test. *, *p* < 0.05, **, *p* < 0.01, ***, *p* < 0.001 compared to control, b, *p* < 0.05, compared with NTX. @, *p* < 0.05, compared with the rest of the groups. (Figure S3: Original gel blots).

Since the beta-adrenergic and the opioidergic agents decreased the breast cancer cell viability, we determined if the effect is associated with the changes in cell cycle arrest [28]. Treatment with 100 µM NTX or PRO alone for 24 h reduced the number of all three cell lines in G0/G1 phase, produced no significant changes in the number in S phase, but increased the number of these cells in G2/M phase (Figure 4A–C and Figure S2A–C). The largest effect on G2/M shift was observed following treatment with NTX + PRO.

To verify the mechanism of G2/M phase arrest induced by NTX, PRO, and NTX + PRO combination, we compared the levels of G2/M phase regulatory proteins (CDK1 and cyclin B) by Western blot analyses. The results show that after a 24 h treatment of NTX, PRO, and NTX + PRO, the levels of CDK1 and cyclin B were significantly downregulated in all three breast cancer cell lines (Figure 4D–L). The downregulation of CDK1 and cyclin B is more pronounced in the NTX + PRO combination. Further, to confirm the effect of NTX, PRO, and NTX + PRO on CDK activity, the phosphorylation status of CDK1 was analyzed. Compared with the control, the phosphorylation of CDK1 (Thr-161) was significantly increased in cells treated with NTX, PRO, and NTX + PRO combination after 24 h treatment (Figure 4G–I). Together, these results suggested that treatment with NTX, PRO, and NTX + PRO downregulated the expression of cyclin B1, CDK1, and the phosphorylation of CDK1, which caused G2/M phase cell cycle arrest without displaying sub-G1 populations in all the breast cancer cells.

### 3.4. Effects on Apoptotic Regulatory Protein Levels and Signaling Pathway in Cancer Cells

**Figure 5:**
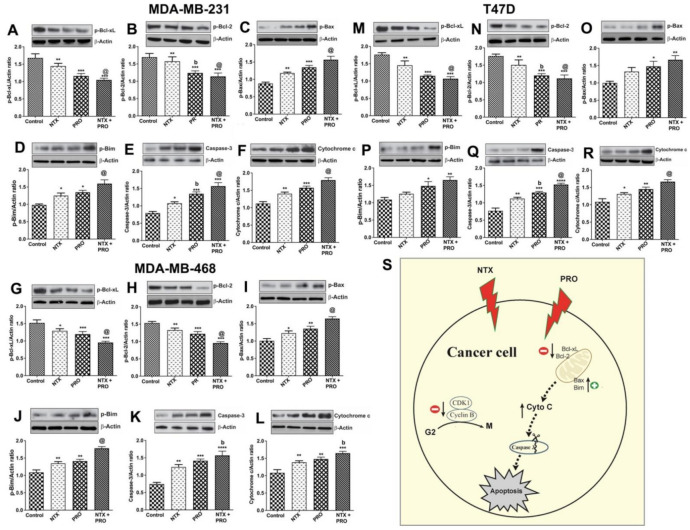
PRO and NTX modulate levels of apoptosis-related protein in MDA-MB-231, MDA-MB-468, and T47D cells. Cells were treated with a 100-μM concentration of the drugs alone or in combination or vehicle alone for 48 h. After the treatment period, cells were extracted and used for Western blot analysis of anti-apoptotic p-Bcl-xL (**A**,**G**,**M**) and p-Bcl2 (**B**,**H**,**N**), effector proteins such as p-Bax (**C**,**I**,**O**), pro-apoptotic molecule p-Bim (**D**,**J**,**P**), effector caspase 3 (**E**,**K**,**Q**) and cytochrome c (**F**,**L**,**R**) levels. Representative blots are presented on the top and mean densitometric values are presented as ratio of β-actin in the histograms. Data presented are mean ± SEM (*n* = 5–6 samples/group) and were analyzed using one-way ANOVA with the Newman–Keuls multiple comparisons post hoc test. *, *p* < 0.05, **, *p* < 0.01, ***, *p* < 0.001, ****, *p* < 0.0001 compared to control, b, *p* < 0.05, compared with NTX. @, *p* < 0.05, compared with the rest of the groups. Schematic illustration of the proposed mechanism of action of naltrexone and propranolol on induction of apoptosis in human breast cancer cells (**S**). (Figure S3: Original gel blots).

Because NTX and PRO promote breast cancer cells to undergo cell cycle arrest in G2/M, the possibility arises that these cells may be undergoing cellular death [28]. The breast cancer cell lines were treated with a single drug or a drug combination for 48 h at 100-μM dose and cell lysate was used for the immunoblotting of various anti-apoptotic BCL-2 family isoforms [29] and proapoptotic caspase 3 and cleaved caspase 3 (CC3) [30] levels. Our immunoblot results showed that the anti-apoptotic BCL-2 family isoforms such as Bcl-xL (Figure S4A–C), p-Bcl-xL (Figure 5A,G,M), Bcl-2 (Figure S4D–F), and p-Bcl-2 (Figure 5B,H,N) were downregulated by NTX, PRO, and NTX + PRO treatment, whereas the treatment of PRO or NTX or a combination of these drugs increased the levels of effector proteins such as Bax (Figure S4G–I) and p-Bax (Figure 5C,I,O) and pro-apoptotic molecules such as p-Bim (Figure 5D,J,P), and also effector caspase 3 (Figure 5E,K,Q) and cleaved caspase 3 (CC3; Figure S4J–L). Importantly, NTX, PRO, and NTX + PRO treatment increased the levels of cytochrome c (Figure 5F,L,R), which is known to play an important role in initiating apoptosis. The effects of these drugs on apoptotic regulatory proteins were enhanced when they were combined compared to when they were given singly by inducing mitochondria-mediated apoptosis. We propose that in breast cancer cells, NTX and PRO treatment causes G2/M phase cell cycle arrest by the downregulation of CDK1, the phosphorylation of CDK1 (activity) and cyclin B1 levels, the upregulation of pro-apoptotic p-Bim and effector proteins such as Bax and p-Bax, and increased levels of effector caspase 3 and CC3. Treatment of NTX and PRO causes increased levels of cytochrome c, thereby causing apoptosis (Figure 5S).

### 3.5. Effect on the Growth of Cell Line-Derived Tumor Xenograft

**Figure 6:**
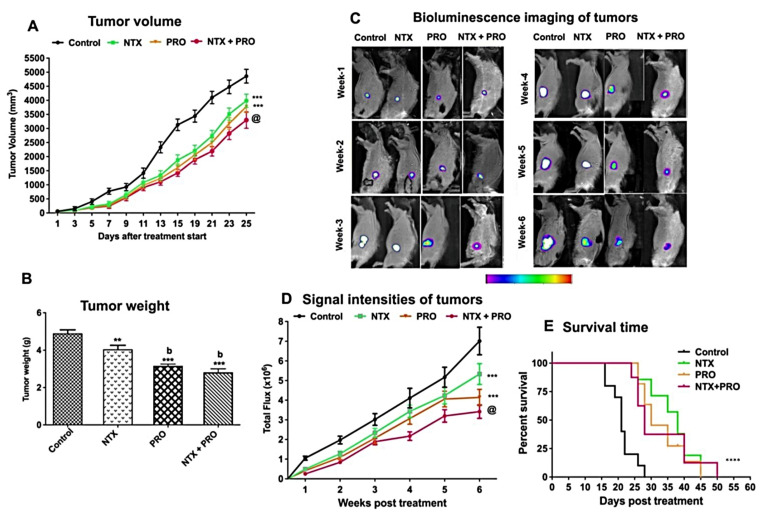
PRO and NTX decrease tumor growth and increase survival in MDA-MB-231-cell-derived xenografts in nude rats. (**A**) Changes in the tumor volume following drug treatments. Values are mean ± SEM (*n* = 8/group). Comparison of tumor volume between groups was performed by two-way ANOVA with Bonferroni’s multiple comparisons test. ***, *p* < 0.001 vs. control. @, *p* < 0.001, compared with the rest of the groups. (**B**) Tumor weight (mean ± SEM; *n* = 5/group) of control and drug-treated rats. Comparison of tumor weight between groups was analyzed by one-way ANOVA with the Newman–Keuls post hoc test. **, *p* < 0.01, ***, *p* < 0.001 vs. control. b, *p* < 0.01, compared with NTX. (**C**) Representative photograph of nude rats injected with MDA-MB 231/Luc-GFP cells and treated with various drug combinations and monitored for a period of 6 weeks by using bioluminescence imaging. Relative intensities of emitted light are represented as a pseudocolor image ranging from purple (least intense) to white (most intense), generated in living image and superimposed onto the grayscale reference image. (**D**) The signal intensity (SI) is expressed as photon flux (photo/sec) from the tumor (mean ± SEM; *n* = 6/group). Comparison of photon flux between groups was performed by two-way ANOVA with Bonferroni’s multiple comparisons test. ***, *p* < 0.001 vs. control. @, *p* < 0.001, compared with the rest of the groups. (**E**) Effects on survival time (days after tumor cell injection) of nude rats inoculated with MDA-MB-231 cells. Kaplan–Meier survival analysis was used to test significant differences between survival curves and mean survival time for rats from each group. Multiple comparisons of survival curves were performed with the Log-rank (Mantel–Cox) test (****, *p* ≤ 0.001 compared to control).

Our in vitro studies using three different breast cancer cell lines showed similar growth inhibitory effects of NTX and PRO; therefore, we limited our in vivo studies by using only MDA-MB-231 cell xenografts in nude rats. Tumor growth as determined by tumor volume was reduced by the treatment with all the drugs tested (Figure 6A). The drug effect on tumor growth was more pronounced when combined than when given alone. Data of tumor wet weight also show that these agents were effective in reducing tumor weight (Figure 6B).

To monitor the effect of drugs on tumor shrinkage/growth in real time, we used MDA-MB 231/Luc-GFP cells in a subcutaneous flank tumor model for bioluminescence imaging [31] in nude rats. We observed that the bioluminescent intensity of the tumor in the control group generally showed an increasing trend similar to that of the tumor volume (Figure 6A,C,D). The combination therapy (NTX + PRO) resulted in a decrease in both luminescent signals and bioluminescent intensity over the 6-week period of treatment.

The determination of survival analysis of MDA-MB-231 cell xenografts using the Kaplan–Meier method [32] shows that the life span of control animals was about 28 days, and the animals treated with a single drug, NTX or PRO, had mean life spans of 45 and 40 days, respectively (Figure 6E). The combination of NTX + PRO improved the survival of animals to 50 days.

### 3.6. Effect on Cell Mitosis, Cell Proliferation, Cellular Apoptosis, and Epithelial–Mesenchymal Transition in Tumor Xenograft

**Figure 7:**
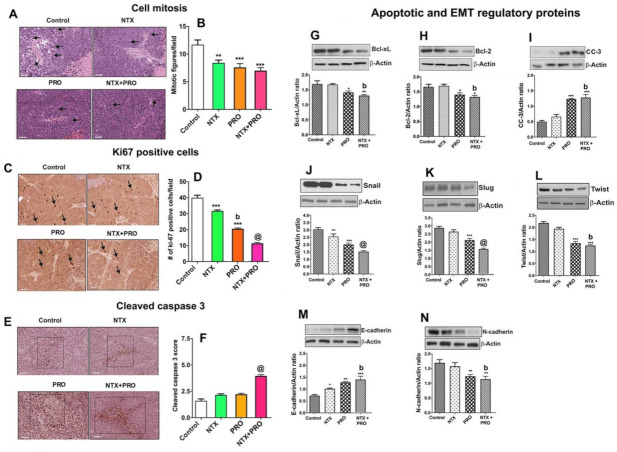
Treatment with PRO or NTX decreases cell mitosis, cell proliferation, cellular apoptosis, and epithelial–mesenchymal transition (EMT) in tumor xenografts. Drug-treated MDA-MB-231-cell-derived xenografts in nude rats were collected and employed for histological determination of cell mitosis (**A**,**B**); immunohistochemical measurements of Ki67 (**C**,**D**) and cleaved caspase 3 (**E**,**F**); and Western blot measurements of Bcl-xL (**G**), Bcl-2 (**H**), and cleaved caspase 3 (**I**); Snail (**J**), Slug (**K**), and Twist (**L**); and E-cadherin (**M**) and N-cadherin (**N**). Changes in tumor histopathology and protein immunohistochemistry are shown by presenting representative pictures (arrows are used to denote the cellular changes whereas a dotted square is used to show the area of intensity). Images represented as 20× magnification with 50 μm scale bars. Histological changes were counted by densitometry and presented in histograms. Data are means ± SEM obtained from 3 serial sections of 5 to 6 animals in each group. Western blot analysis of protein levels was measured by densitometry and presented as ratio of β-actin, a house-keeping protein, in histograms. Data presented are mean ± SEM (*n* = 5–6 animals/group) and were analyzed using one-way ANOVA with the Newman–Keuls multiple comparisons test. *, *p* < 0.05, **, *p* < 0.01, ***, *p* ≤ 0.001 compared to control, b, *p* < 0.01, compared with NTX. @, *p* < 0.001, compared with the rest of the groups. (Figure S3: Original gel blots).

Histological examination of xenograft tumors from each group showed that control animals had healthy intact tumor cells with the presence of many mitotic figures in the tumor. Xenograft tumors from beta-adrenergic and opioidergic-treated animals had a decreased number of mitotic figures. The combination of NTX and PRO treatment showed the most prominent reduction in mitotic figures (Figure 7A,B). We also used an immunostaining procedure to evaluate the number of ki-67-positive cells as a marker for cell proliferation [33] and CC3 as an indicator of apoptotic bodies [34] in each group. Data show that control animals had a higher number of proliferating cells (Figure 7C,D), but they had a lower number of apoptotic cells in the center of the tumor (Figure 7E,F). Single-drug treatment moderately decreased the ki-67 levels in the tumor while combined-drug treatment markedly decreased the ki-67 levels in the tumor. Conversely, combined-drug treatments, particularly double-drug treatments, markedly increased the CC3 levels in the tumor.

A growing body of research shows that Bcl-xL and Bcl-2 act as pro-survival factors [29], whereas activated (cleaved) forms of caspases, such as CC3, participate in executing apoptosis within a cell [30]. Data show the treatment with NTX or PRO alone or in combination decreased the levels of Bcl-xL and Bcl-2 while they increased the level of CC3 (Figure 7G–I). The most potent effects of these drugs on the levels of these proteins in the tumor were observed when three drugs were combined.

We also measured the levels of epithelial–mesenchymal transition (EMT) factors (Snail, Slug, and Twist), epithelial (E-cadherin) markers, and mesenchymal (N-cadherin) markers in tumor tissues since these proteins are known to play important roles in acquiring the invasive and metastatic property of cancer cells [35]. Immunoblotting results of EMT factors and markers indicated that single-drug (moderately by NTX and strongly by PRO) treatment reduced the levels of Snail (Figure 7J), Slug (Figure 7K), and Twist (Figure 7L), with a concomitant decrease in the level of N-cadherin (Figure 7N) and an increase in the level of E-cadherin (Figure 7M) in tumors. Notably, this trend of modulation in these proteins showed an additive effect in the tumors of animals treated with combination drugs.

### 3.7. Effects of Drugs on Immune Cell Functions in the Tumor Xenograft Host

**Figure 8:**
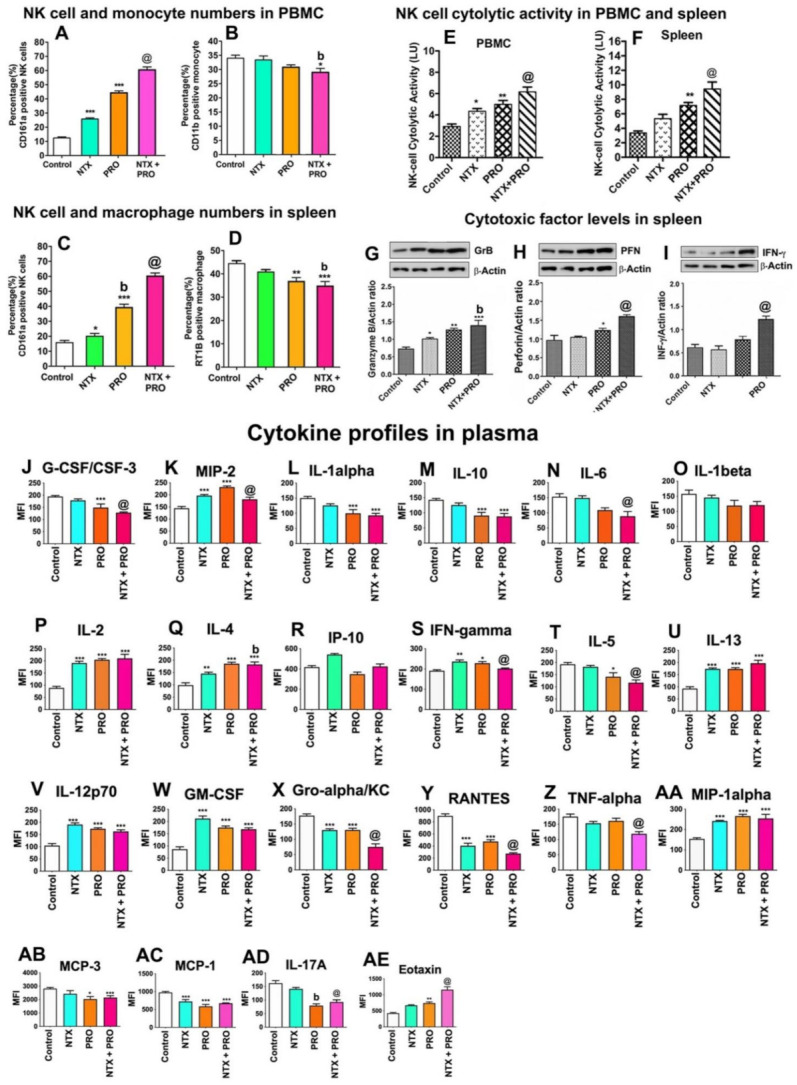
Beta-adrenergic and opioidergic agents enhance immune cell function in breast cancer cell xenografted animals. PBMC samples and spleen samples of these rats were employed for flow cytometric measurements of CD161a^+^ NK cell (**A**,**C**), CD11b^+^ monocyte (**B**), and RT1B^+^ macrophage cell numbers (**D**); both PBMC and spleen samples of these rats were used for NK cell cytolytic activity (**E**,**F**); spleens of these animals were also used for cytotoxic proteins Granzyme B (GzB; **G**), perforin (PFN; **H**) and IFN-γ (**I**); and plasma was used for cytokine measurements (**J**–**AE**). Each fluorescence histogram’s top panel shows the percentages of immune cells in control and treated rats. Data are shown for isotype control (gray) and specific cell surface marker (blue). The bar graphs (below) show the mean ± SEM percentage of immune cells. Plasma samples were used for measurements of cytokines using Luminex Multiplex Immunoassay. Data are expressed as mean ± SEM (*n* = 5–6). Statistical significance was determined using one-way ANOVA with the Newman–Keuls multiple comparisons test. *, *p* < 0.05, **, *p* < 0.01, ***, *p* ≤ 0.001 compared to control. @, *p* < 0.05, compared with NTX. @, *p* < 0.05, compared with the rest of the groups. (Figure S3: Original gel blots).

The tumor xenograft study was conducted in athymic nude rats, and therefore the major immune defense mechanism against cancer is controlled by the innate immune system in this host [36]. PBMCs and spleen tissue of xenografted rats treated with different treatments and control animals were used for screening immune cell populations by flow cytometry to quantitate the percentage (%) of CD161a+ NK cells in PBMCs (Figure 8A), CD11b+ monocytes in PBMCs (Figure 8B), CD161a+ NK cells in the spleen (Figure 8C), and RT1B+ macrophages in the spleen (Figure 8D). Treatment with NTX or PRO alone increased NK cell numbers in PBMCs and the spleen. The effects of these drugs were potentiated when drugs were combined. In contrast, monocyte numbers in PBMCs were decreased following NTX + PRO treatments, and macrophage numbers in the spleen were reduced following PRO and NTX + PRO treatments.

The changes in the cytolytic activity of PBMC-derived and spleen-derived NK cells following the drug treatments are shown in Figure 8E,F. NK cell cytolytic activity was elevated by PRO treatment, and this effect of PRO was magnified when combined with NTX. Measurements of the levels of cytolytic proteins (granzyme B, perforin, and IFN-γ) in NK cells enriched from the spleen of the host animal also showed stimulatory effects of beta-adrenergic and opioidergic drugs (Figure 8G–I). The combination of NTX with PRO produced the maximal effects on these cytotoxic proteins in NK cells of the spleen.

Cytokines are known to be critical autocrine and paracrine factors in tumor development, which are secreted into the tumor microenvironment to recruit and activate various inflammatory cells for promotion evasion from immune destruction [37]. Measurements of cytokine levels in the plasma of these rats indicated that PRO inhibited a large number of inflammatory cytokines and chemokines such as G-CSF/CSF-3, IL-1 alpha, IL-10, IL-6, IL-5, Gro-α, TNF-α, MCP-3, and IL-17A (Figure 8J,L–N,T,X,Z,AB,AD). The combination of PRO with NTX showed a greater inhibition of these inflammatory cytokines and chemokines, especially IL-6 (Figure 6N), RANTES, and MCP-1 (Figure 8Y,AC). On the contrary, the different combination treatments of PRO and NTX increased the concentration in the plasma of cytokines involved in the response of NK cells: IL-2, IL-4, IL-13, IL-12p70 (Figure 8P,Q,U,V), and IFN-γ (Figure 8S), with the higher mean level reached for the combined treatment group. Finally, the macrophage inflammatory proteins MIP-2 and MIP-1α (Figure 8K,AA), GM-CSF, and Eotaxin (Figure 8W,AE) were shown to be elevated in most of the treatment groups, with the highest mean level achieved by double-drug treatments.

**Figure 9:**
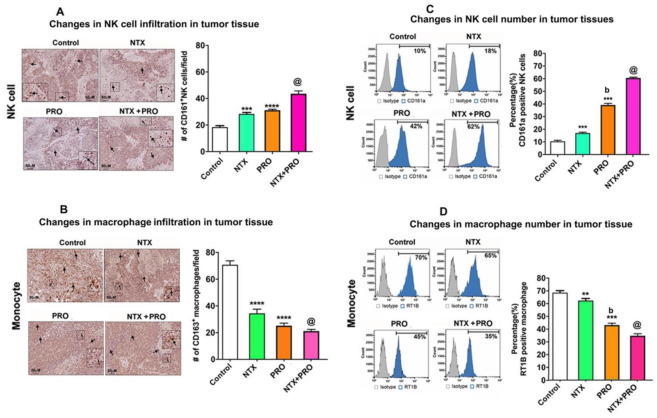
Beta-adrenergic and opioidergic agents alter immune cells in the tumor in breast cancer cell xenografted rats. (**A**,**B**) Left: Representative microscopic pictures of immunocytochemical staining demonstrating infiltration of CD161^+^ NK cells and CD163^+^ macrophages in tumor tissues in various treatment groups. Arrows indicate the staining of NK cells and macrophages. Scale bars are 50-µm. Inset in each figure shows a magnified view of representative NK cells and macrophages. Right: Histograms showing numbers of tumor infiltrated NK cells and macrophages quantified from six different fields and presented as mean ± SEM (*n* = 5–6 animals/group). (**C**,**D**) Left: Fluorescence histograms showing the percentages of CD161a-expressing NK cells and MHC class II CRT1B-expressing macrophages from tumor tissues of control and treated rats. Data are shown for isotype control (gray) and specific cell surface marker (blue). Right: The bar graphs show the mean ± SEM (*n* = 5–6 animals/group) percentage NK cells and macrophages as determined from single-color (parameter) flow cytometry histogram. **, *p* < 0.01, ***, *p* <0.001, ****, *p* <0.0001 compared to control, b, *p* < 0.05, compared with NTX treatment. @, *p* < 0.05, compared with the rest of the treatment groups.

Further, to understand how these drugs modulate the immune cell populations in the tumor, we used immunocytochemical analysis of NK cell and macrophage infiltration in the tumor tissues (Figure 9A,B) and flow cytometry analyses of NK cell and macrophage numbers in tumor tissues (Figure 9C,D). Both immunocytochemical data and flow cytometry analysis show that NTX or PRO alone or in combination increased the percentage of CD161+ NK cells while they decreased the percentage of CD163+ macrophages at varying degrees in the tumor tissues. We also observed the maximal effects on immune cell migrations in the tumor by the treatment of NTX + PRO.

## 4. Discussion

The data presented here add to the evidence that supports the use of PRO as an anti-metastatic agent in breast cancer, particularly effective in the neo-adjuvant period or as a perioperative therapy [38]. PRO use in early-stage breast cancer is associated with a reduction in ki67 indices [39]. Data from a neoadjuvant exposure trial in breast cancer on PRO + COX2 inhibitor also showed effects on EMT and the immune microenvironment [38]. Additionally, significant innervation of sympathetic nerves has been documented in human breast cancer [40]. However, PRO is a non-specific β2-adrenergic blocker and acts via GPCRs, which are known to heterodimerize with closely related members, resulting in the modulation of their functions [41]. Hence, there remains a potential for PRO to combine with other agents known to act via GPCRs to increase its antitumor effectiveness. It has been shown that opioid receptors can form heteromeric complexes with B2ARs and affect the signal transduction of B2ARs [21]. Here, we showed that MOR agonist NTX potentiated B2AR antagonist PRO effects on breast cancer cells. The heteromeric interaction between B2AR and MOR may also explain why PRO is more effective in reducing tumor growth, preventing metastasis, and increasing the survival of the animal with breast cancer when it is combined with NTX. It was identified that PRO significantly stimulates NK cell functions, and NTX promotes PRO effects on expanded NK cells from the spleens and PBMCs of tumor xenografted animals. Previously, it has been shown that the stimulatory guanine-nucleotide-binding protein GS coupled to B2AR is involved in transducing signals that inhibit NK cell lysis of tumor cells [42]. Additionally, it has been shown that opioid receptors constitutively produce and function in rat spleens and PBMC-derived NK cells [43] and human PBMC-derived NK cells [44]. In the rat, NTX suppresses MOR to stimulate PBMC- and spleen-derived NK cell cytolytic functions [43]. Thus, the heteromeric interaction between B2AR and MOR may also explain why PRO is more effective in inducing NK cell cytolytic functions when it is combined with NTX. However, further study involving physical interaction between these receptors is needed to establish the role of the heteromeric interaction between B2AR and MOR in regulating breast cancer and NK cell functions.

We also observed that PRO and NTX increased levels of cytokines involved in the modulation of NK cells, while they reduced levels of Th1 inflammatory cytokines in blood in our xenograft animals. These data are in agreement with the findings that PRO increases cytokines IL-2, IL-4, IL-12, IL-17, IL-12p70, and IFN-γ in animal models of breast cancer [45]. In this study, we confirmed the activation effect of PRO on these cytokines and demonstrated that the combination of PRO with NTX potentiated the increased levels of these cytokines. Many pro-inflammatory cytokines are overexpressed in breast cancer and associated with a poor prognosis and a large impact on the latest stage of cancer, such as angiogenesis and metastasis [46,47]. We showed in this study a diminution of the plasma level of many pro-inflammatory cytokines that could suggest a less aggressive cancer profile following PRO and/or NTX treatments. Because the xenograft animal models we used were athymic rats and they lack T-cell function, the effects of these drugs on Th2 cytokines cannot be properly determined in this animal model. However, the strong inhibitory effects of tested drugs we observed on plasma levels of inflammatory cytokines are in agreement with previous findings that these agents reduce pro-inflammatory cytokines [48,49].

We found an increase in the number of antitumor NK cells but a decrease in the number of protumor monocytes/macrophages in the spleen, PBMCs, and tumor tissues in association with a reduction in tumor volume in PRO- and/or NTX-treated animals. After their recruitment into the tumor tissue, monocytes are known to differentiate into tumor-associated macrophages (TAM), a very heterogeneous cell population in terms of phenotype and protumor function, and support tumor initiation, local progression, and distant metastasis [50]. Classically activated macrophages (M1), following exposure to interferons, have antitumor activity and elicit tissue destructive reactions; however, in response to inflammatory cytokines, macrophages undergo alternative activation (M2) and gain protumor activity [51]. Therefore, PRO and NTX significantly influenced immune cell infiltration and tumor invasion to promote antitumor function.

We used athymic (nude) rats for xenografts in this study. These rats are T-cell-deficient but are NK-cell-intact athymic nude, and therefore the major immune defense mechanism against cancer is controlled by NK cells in this host [36]. NK cells are a key group of immune cells that play an important role in controlling the tumor growth [52]. NK cells are also key targets of NTX [23] and PRO [53,54].

## 5. Conclusions

In summary, we demonstrated in this study that the treatment of PRO and NTX, individually or in combination, inhibited the cell growth, colony formation, migration, invasion, and cell cycle progression of MDA-MB-231, MDA-MB-468, and T47D at varying degrees in vitro. The antitumor activities of PRO and NTX increased when combined compared to single-drug treatment. In the MDA-MB-231 cell xenograft nude rat model, it was identified that these drugs alone, and more potently when combined, reduced tumor growth and increased animal survivability. The antitumor activities of these drugs were associated with increased cell growth arrest, elevated levels of apoptotic proteins, reduced production of epithelial–mesenchymal transition factors in tumor cells, the activation of cytolytic functions of NK cells, increased infiltration of NK cells in the tumor, and reduced inflammatory cytokine levels in plasma. These data suggest that the combined treatments of PRO and NTX produce impressive antitumor effects in the preclinical breast cancer model. Therefore, combining PRO and NTX may have potential therapeutic value for the treatment of breast cancer.

## Data Availability

All relevant data are included in this manuscript. Any requests for materials will be fulfilled upon request through appropriate means.

## Supplementary Materials

The following are available online at https://www.mdpi.com/article/10.3390/cancers13194858/s1, Figure S1: Beta-adrenergic and opioidergic agents suppress breast tumor cells’ clonogenic behavior, cell migration, and invasion. Figure S2: Beta-adrenergic and opioidergic agents increase cell growth arrest in breast cancer cell lines. Figure S3: Original gel blots. Figure S4: Beta-adrenergic and opioidergic agents alter the levels of apoptotic regulatory protein in breast cancer cell lines. Table S1: List of antibodies used for immunocytochemistry and Western blot analysis and cell lines, Table S2: List of antibodies and dyes used in flow cytometry, Table S3: List of isotype control and beads used in flow cytometry, Table S4: Combination index (CI) values for NTX with PRO.

## References

[1] National Cancer Institute Cancer Stat Facts: Female Breast Cancer. https://seer.cancer.gov/statfacts/html/breast.html.

[2] Hwang K.T., Kim J., Jung J., Chang J.H., Chai Y.J., Oh S.W., Oh S., Kim Y.A., Park S.B., Hwang K.R. (2019). Impact of breast cancer subtypes on prognosis of women with operable invasive breast cancer: A population-based study using SEER database. Clin. Cancer Res..

[3] D’Arcy M., Fleming J., Robinson W.R., Kirk E.L., Perou C.M., Troester M.A. (2015). Race-associated biological differences among Luminal A breast tumors. Breast Cancer Res. Treat..

[4] Burugu S., Asleh-Aburaya K., Nielsen T.O. (2017). Immune infiltrates in the breast cancer microenvironment detection, characterization and clinical implication. Breast Cancer.

[5] Antoni M.H., Lutgendorf S.K., Cole S.W., Dhabhar F.S., Sephton S.E., McDonald P.G., Stefanek M., Sood A.K. (2006). The influence of bio-behavioural factors on tumour biology: Pathways and mechanisms. Nat. Rev. Cancer.

[6] Webster J.I., Tonelli L., Sternberg E.M. (2002). Neuroendocrine regulation of immunity. Annu. Rev. Immunol..

[7] Smyth M.J., Cretney E., Kershaw M.H., Hayakawa Y. (2004). Cytokines in cancer immunity and immunotherapy. Immunol. Rev..

[8] Herberman R.B. (1984). Possible role of natural killer cells and other effector cells in immune surveillance against cancer. J. Investig. Dermatol..

[9] Lavandero S., Donoso E., Sapag-Hagar M. (1985). Beta-adrenergic receptors in rat mammary gland. Biochem. Pharmacol..

[10] Vandewalle B., Revillion F., Lefebvre J. (1990). Functional beta-adrenergic receptors in breast cancer cells. J. Cancer Res. Clin. Oncol..

[11] Sloan E.K., Priceman S.J., Cox B.F., Yu S., Pimentel M.A., Tangkanangnukul V., Arevalo J.M., Morizono K., Karanikolas B.D., Wu L. (2010). The sympathetic nervous system induces a metastatic switch in primary breast cancer. Cancer Res..

[12] Barron T.I., Connolly R.M., Sharp L., Bennett K., Visvanathan K. (2011). Beta blockers and breast cancer mortality: A population-based study. J. Clin. Oncol..

[13] Barron T.I., Sharp L., Visvanathan K. (2012). Beta-adrenergic blocking drugs in breast cancer: A perspective review. Ther. Adv. Med. Oncol..

[14] Lutz P.E., Kieffer B.L. (2013). Opioid receptors: Distinct roles in mood disorders. Trends Neurosci..

[15] De Kloet E.R., Reul J.M. (1987). Feedback action and tonic influence of corticosteroids on brain function: A concept arising from the heterogeneity of brain receptor systems. Psychoneuroendocrinology.

[16] Kreek M.J. (2001). Drug addictions: Molecular and cellular endpoints. Ann. N. Y. Acad. Sci..

[17] Lovallo W.R., Enoch M.A., Acheson A., Cohoon A.J., Sorocco K.H., Hodgkinson C.A., Vincent A.S., Glahn D.C., Goldman D. (2015). Cortisol stress response in men and women modulated differentially by the mu-opioid receptor gene polymorphism OPRM1 A118G. Neuropsychopharmacology.

[18] Cieślińska A., Sienkiewicz-Szłapka E., Kostyra E., Fiedorowicz E., Snarska J., Wroński K., Tenderenda M., Jarmołowska B., Matysiewicz M. (2015). μ-Opioid receptor gene (OPRM1) polymorphism in patients with breast cancer. Tumour Biol..

[19] Cronin-Fenton D. (2019). Opioids and breast cancer recurrence. Curr. Opin. Support. Palliat. Care.

[20] Couto R.D., Fernandes B.J.D. (2021). Low Doses Naltrexone: The Potential Benefit Effects for its Use in Patients with Cancer. Curr. Drug Res. Rev..

[21] Jordan B.A., Trapaidze N., Gomes I., Nivarthi R., Devi L.A. (2001). Oligomerization of opioid receptors with beta 2-adrenergic receptors: A role in trafficking and mitogen-activated protein kinase activation. Proc. Natl. Acad. Sci. USA.

[22] Dai X., Cheng H., Bai Z., Li J. (2017). Breast cancer cell line classification and its relevance with breast tumor subtyping. J. Cancer.

[23] Sarkar D.K., Sengupta A., Zhang C., Boyadjieva N., Murugan S. (2012). Opiate antagonist prevents μ- and δ-opiate receptor dimerization to facilitate ability of agonist to control ethanol-altered natural killer cell functions and mammary tumor growth. J. Biol. Chem..

[24] Chou T.C., Talalay P. (1984). Quantitative analysis of dose-effect relationships: The combined effects of multiple drugs or enzyme inhibitors. Adv. Enzyme. Regul..

[25] Franken N.A., Rodermond H.M., Stap J., Haveman J., van Bree C. (2006). Clonogenic assay of cells in vitro. Nat. Protoc..

[26] Justus C.R., Leffler N., Ruiz-Echevarria M., Yang L.V. (2014). In vitro cell migration and invasion assays. J. Vis. Exp..

[27] Zhang C., Murugan S., Boyadjieva N., Jabbar S., Shrivastava P., Sarkar D.K. (2015). Beta-endorphin cell therapy for cancer prevention. Cancer Prev. Res..

[28] Nordin N., Yeap S.K., Rahman H.S., Zamberi N.R., Abu N., Mohamad N.E., How C.W., Masarudin M.J., Abdullah R., Alitheen N.B. (2019). In vitro cytotoxicity and anticancer effects of citral nanstructured lipid carrier on MDAMBA-231 human breast cancer cells. Sci. Rep..

[29] Warren C.F.A., Wong-Brown M.W., Bowden N.A. (2019). BCL-2 family isoforms in apoptosis and cancer. Cell Death Dis..

[30] Crowley L.C., Waterhouse N.J. (2016). Detecting cleaved caspase-3 in apoptotic cells by flow cytometry. Cold Spring Harb. Protoc..

[31] Mezzanotte L., Fazzina R., Michelini E., Tonelli R., Pession A., Branchini B., Roda A. (2010). In vivo bioluminescence imaging of murine xenograft cancer models with a red-shifted thermostable luciferase. Mol. Imaging Biol..

[32] Villanueva N.M., Sestelo M., Meira-Machado L. (2019). A method for determining groups in multiple survival curves. Stat. Med..

[33] Juríková M., Danihel L., Polák S., Varga I. (2016). Ki67, PCNA and MCM proteins: Markers of proliferation in the diagnosis of breast cancer. Acta. Histochem..

[34] Porter A.G., Jänicke R.U. (1999). Emerging roles of caspase-3 in apoptosis. Cell Death Differ..

[35] Felipe Lima J., Nofech-Mozes S., Bayani J., Bartlett J.M. (2016). EMT in breast carcinoma—A review. J. Clin. Med..

[36] Hougen H.P. (1991). The athymic nude rat. Immunobiological characteristics with special reference to establishment of non-antigen-specific T-cell reactivity and induction of antigen-specific immunity. APMIS Suppl..

[37] Fasoulakis Z., Kolios G., Papamanolis V., Kontomanolis E.N. (2018). Interleukins associated with breast cancer. Cureus.

[38] Shaashua L., Shabat-Simon M., Haldar R., Matzner P., Zmora O., Shabtai M., Sharon E., Allweis T., Barshack I., Hayman L. (2017). Perioperative COX-2 and β-adrenergic blockade improves metastatic biomarkers in breast cancer patients in a phase-II randomized trial. Clin. Cancer Res..

[39] Montoya A., Amaya C., Belmont A., Diab N., Trevino R., Villanueva G., Rains S., Sanchez L.A., Badri N., Otoukesh S. (2017). Use of non-selective β-blockers is associated with decreased tumor proliferative indices in early stage breast cancer. Oncotarget.

[40] Faulkner S., Jobling P., March B., Jiang C.C., Hondermarck H. (2019). Tumor neurobiology and the war of nerves in cancer. Cancer Discov..

[41] Xiao R.P., Pepe S., Spurgeon H.A., Capogrossi M.C., Lakatta E.G. (1997). Opioid peptide receptor stimulation reverses beta-adrenergic effects in rat heart cells. Am. J. Physiol..

[42] Bimonte S., Barbieri A., Cascella M., Rea D., Palma G., Del Vecchio V., Forte C.A., Del Prato F., Arra C., Cuomo A. (2018). The effects of naloxone on human breast cancer progression: In vitro and in vivo studies on MDA.MB231 cells. Onco. Targets Ther..

[43] Boyadjieva N.I., Chaturvedi K., Poplawski M.M., Sarkar D.K. (2004). Opioid antagonist naltrexone disrupts feedback interaction between mu and delta opioid receptors in splenocytes to prevent alcohol inhibition of NK cell function. J. Immunol..

[44] Pérez Piñero C., Bruzzone A., Sarappa M.G., Castillo L.F., Lüthy I.A. (2012). Involvement of α2- and β2-adrenoceptors on breast cancer cell proliferation and tumour growth regulation. Br. J. Pharmacol..

[45] Ashrafi S., Shapouri R., Shirkhani A., Mahdavi M. (2018). Anti-tumor effects of propranolol: Adjuvant activity on a transplanted murine breast cancer model. Biomed. Pharmacother..

[46] Hollmén M., Karaman S., Schwager S., Lisibach A., Christiansen A.J., Maksimow M., Varga Z., Jalkanen S., Detmar M. (2015). G-CSF regulates macrophage phenotype and associates with poor overall survival in human triple-negative breast cancer. Oncoimmunology.

[47] Dutta P., Sarkissyan M., Paico K., Wu Y., Vadgama J.V. (2018). MCP-1 is overexpressed in triple-negative breast cancers and drives cancer invasiveness and metastasis. Breast Cancer Res. Treat..

[48] Partecke L.I., Speerforck S., Käding A., Seubert F., Kühn S., Lorenz E., Schwandke S., Sendler M., Keßler W., Trung D.N. (2016). Chronic stress increases experimental pancreatic cancer growth, reduces survival and can be antagonized by beta-adrenergic receptor blockade. Pancreatology.

[49] Parkitny L., Younger J. (2017). Reduced pro-inflammatory cytokines after eight weeks of low-dose naltrexone for fibromyalgia. Biomedicines.

[50] Abel A.M., Yang C., Thakar M.S., Malarkannan S. (2018). Natural killer cells: Development, maturation, and clinical utilization. Front. Immunol..

[51] Sica A. (2010). Role of tumour-associated macrophages in cancer-related inflammation. Exp. Oncol..

[52] Wu S.Y., Fu T., Jiang Y.Z., Shao Z.M. (2020). Natural killer cells in cancer biology and therapy. Mol. Cancer.

[53] Hiller J.G., Cole S.W., Crone E.M., Byrne D.J., Shackleford D.M., Pang J.B., Henderson M.A., Nightingale S.S., Ho K.M., Myles P.S. (2020). Preoperative beta-blockade with propranolol reduces biomarkers of metastasis in breast cancer: A phase II randomized trial. Clin. Cancer Res..

[54] Tarr A.J., Powell N.D., Reader B.F., Bhave N.S., Roloson A.L., Carson W.E., Sheridan J.F. (2012). Beta-adrenergic receptor mediated increases in activation and function of natural killer cells following repeated social disruption. Brain Behav. Immun..

