# Supplementary material for: Beta 2 Adrenergic Receptor Antagonist Propranolol and Opioidergic Receptor Antagonist Naltrexone Produce Synergistic Effects on Breast Cancer Growth Prevention by Acting on Cancer Cells and Immune Environment in a Preclinical Model of Breast Cancer
Source: Cancers (Basel). 2021 Sep 28;13(19):4858. doi: 10.3390/cancers13194858 (PMC8508249; doi:10.3390/cancers13194858)
Supplement: Supplementary file 1 [file cancers-13-04858-s001.zip › cancers-1285614-supplementary.pdf]

**Supplementary Information**

**Beta 2 adrenergic receptor antagonist propranolol and opioidergic receptor antagonist naltrexone produce synergistic effects on breast cancer growth prevention by acting on cancer cells and immune environment in a preclinical model of breast cancer.**

Sengottuvelan Murugan, Bénédicte Rousseau, and Dipak K. Sarkar

**Supplementary Figures**

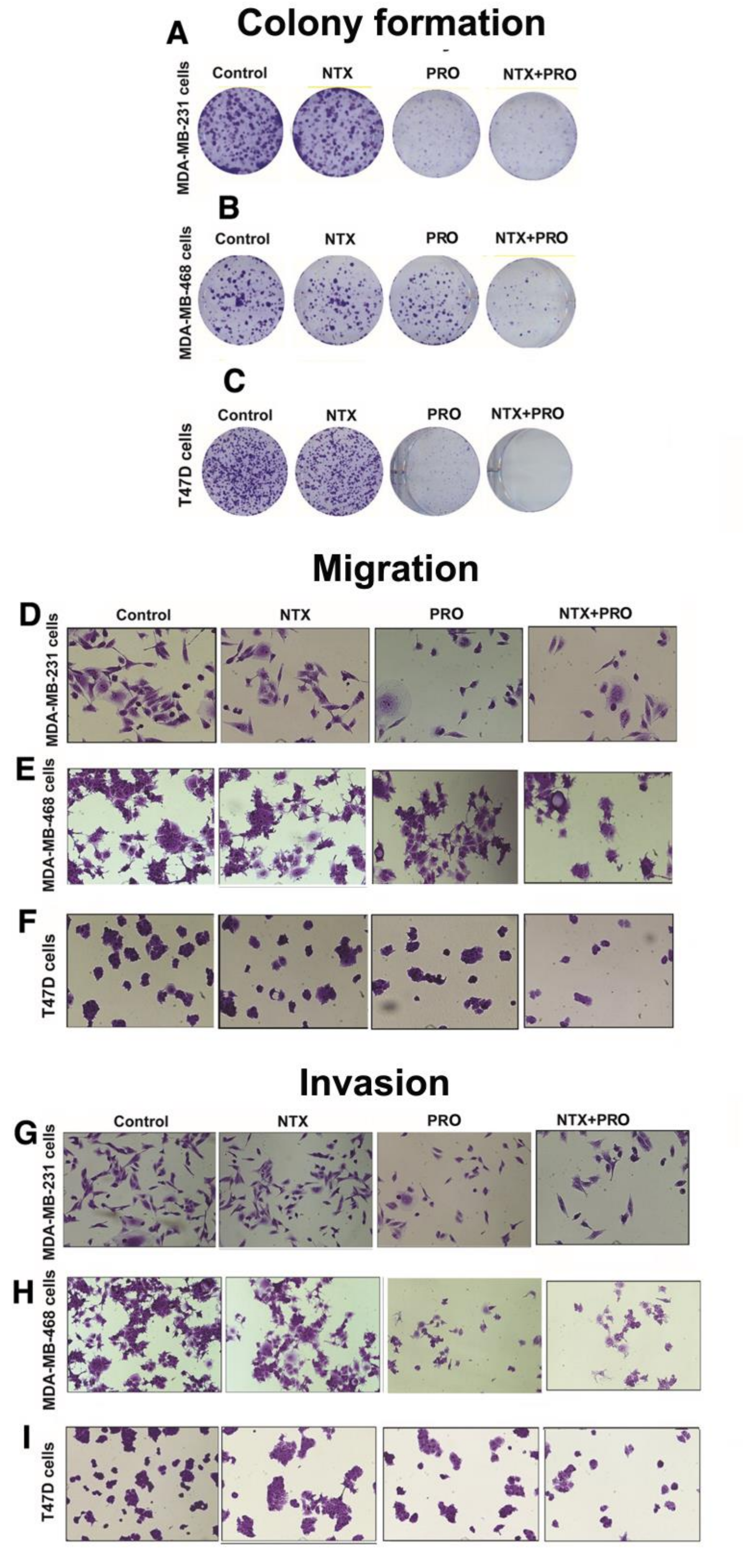


**Figure S1. Beta-adrenergic and opioidergic agents suppress breast tumor cells' clonogenic behavior, cell migration, and invasion**. Representative images of MDA-MB-231 (**A**), MDA-MB-468 (**B**), and T47D (**C**) crystal violet stained cell colonies after 14 days of treatment with a 100 μM dose of PRO and NTX alone or in combination. The crystal violet stained cell colonies were extracted with 10% acetic acid and used for optical density (OD) measurements for determination of colony growth. These data are presented in Fig. 1B-D. Cell migration was determined using transwell migration assay. Cells were stained with 0.5% crystal violet from control or after NTX, PRO, or NTX+PRO treatments as described in materials and methods. Representative images of migrated MDA-MB-231 cells (**D**), MDA-MB-468 cells (**E**), and T47D cells (**F**) are shown. The mean ± SEM values of cell migration data are presented in Figure 1E-G. Cell invasion was tested on a Matrigel™ coated membrane. Cells were treated with the drugs and stained

with 0.5% crystal violet after completion of the test. Representative images are shown here for MDA-MB-231 cells (**G**), MDA-MB-468 cells (**H**), and T47D cells (**I**), and mean ± SEM values of cell invasion data are presented in Figure 1H–J.

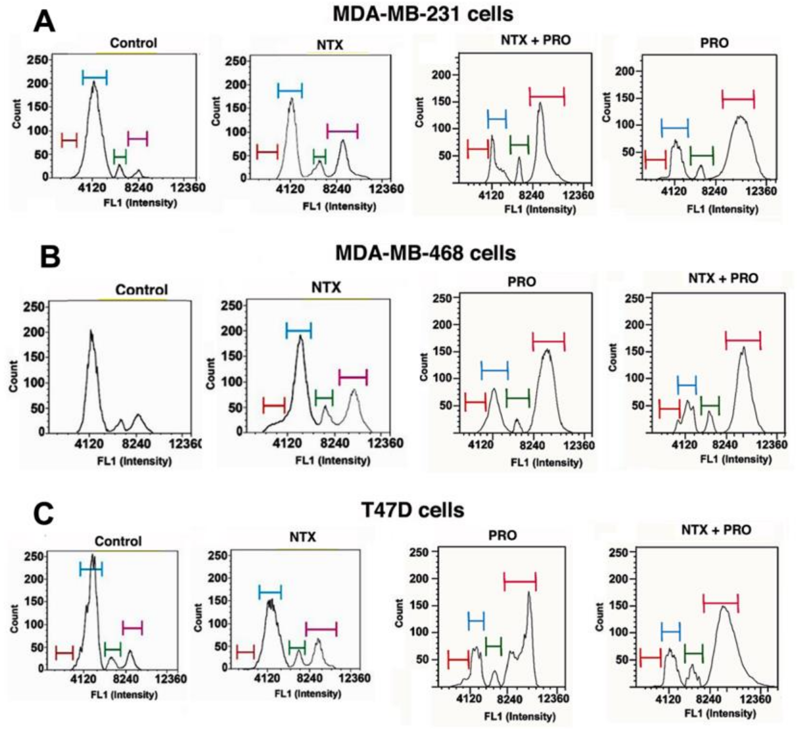


**Figure S2. Beta-adrenergic and opioidergic agents increase the cell growth arrest in breast cancer cell lines.** Cell cycle analysis of MDA-MB-231 (**A**), MDA-MB-468 (**B**), and T47D (**C**) cells following treatments with NTX, PRO, or NTX+PRO were conducted as described in materials and methods. Cells were treated with a 100 μM concentration of the beta-adrenergic and opioidergic drugs alone or in combination or vehicle alone for 24 hours. After the treatment period, cells were stained with PI, analyzed for cell cycle distribution using flow cytometry, and shown as grafts. Proportions of cells in each phase were quantified and shown as histograms in Figure 2A–C.

**Figure S3 Original gel blots (Refer to Figure4,5,7,8):**

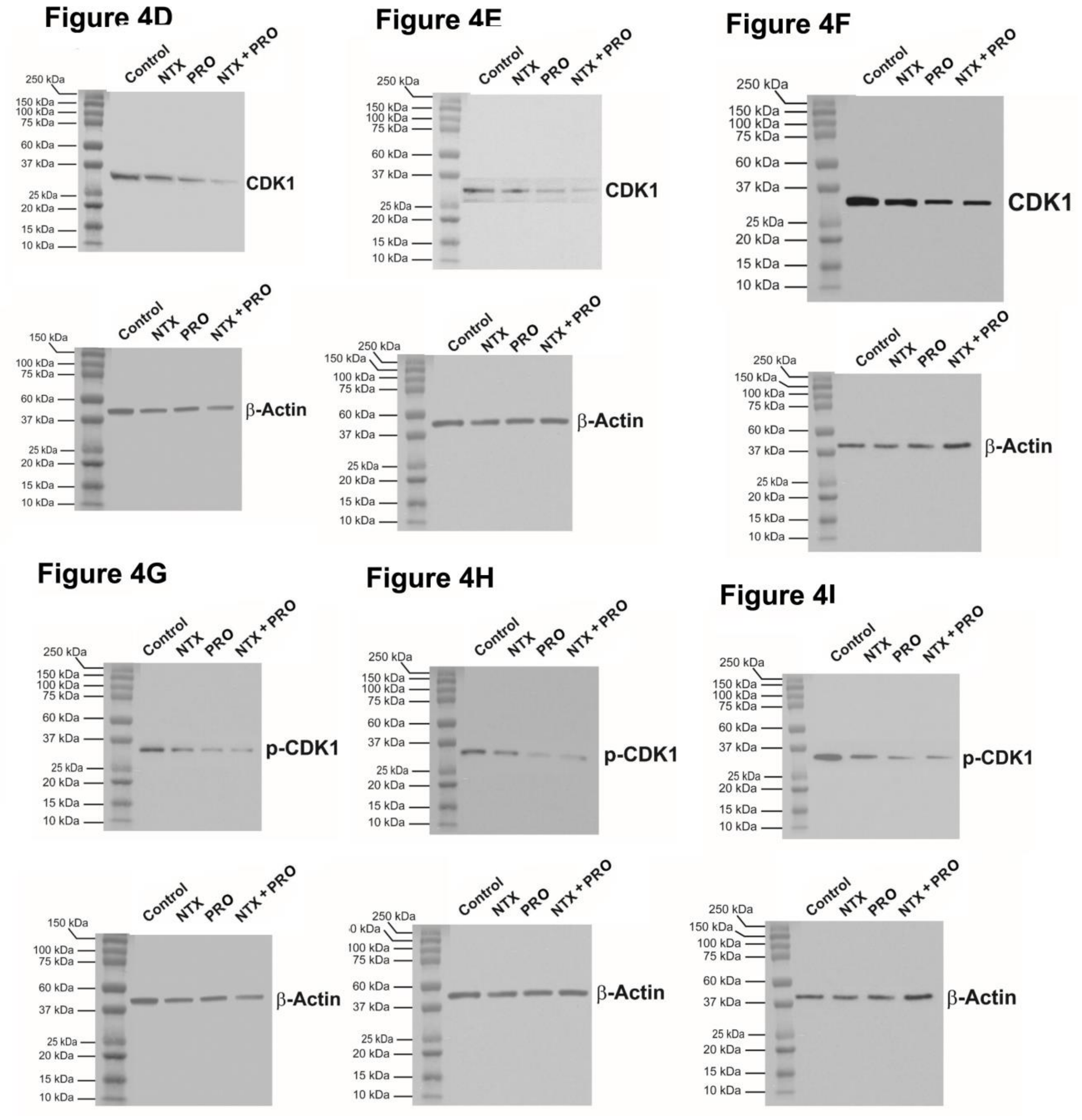

## Figure 4J

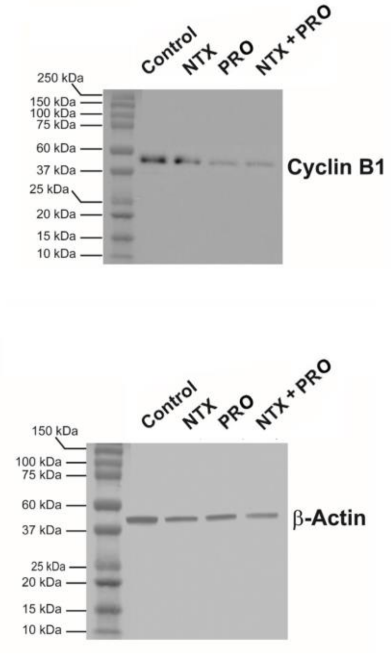


## Figure 4K

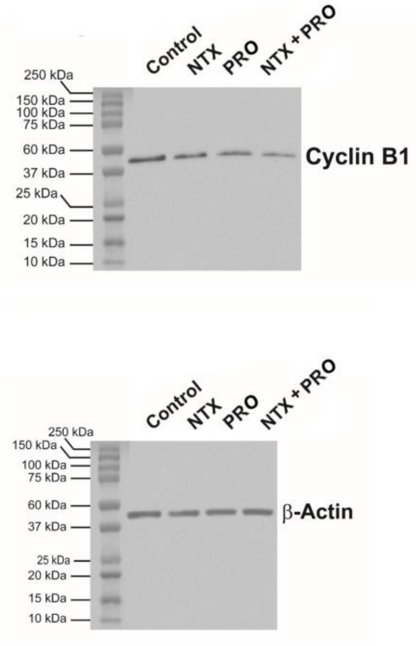


## Figure 4L

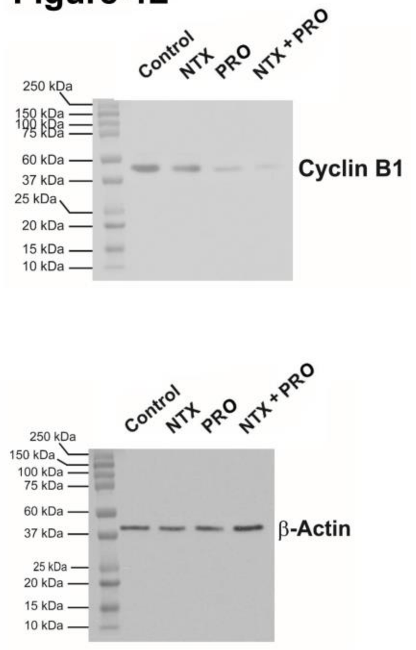

Figure 5A

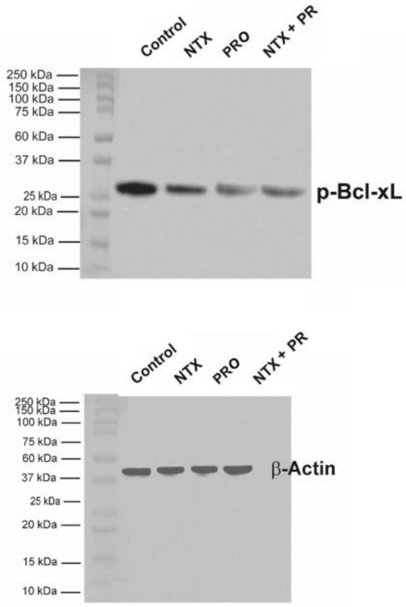


Figure 5G

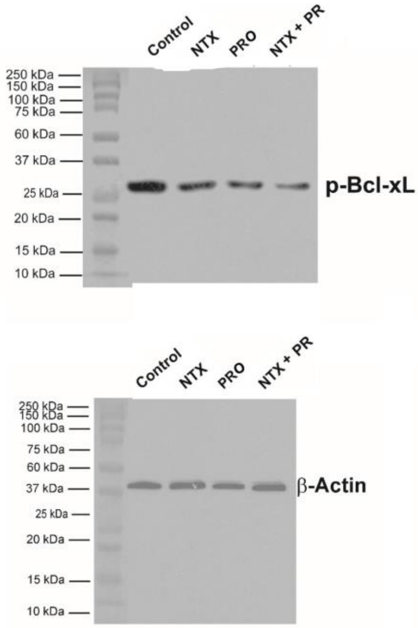


Figure 5M

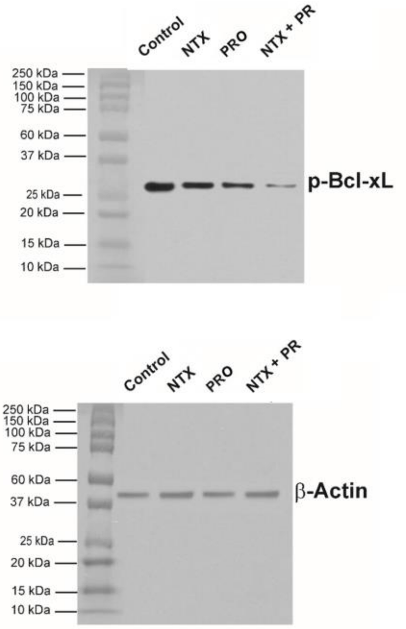


Figure 5B

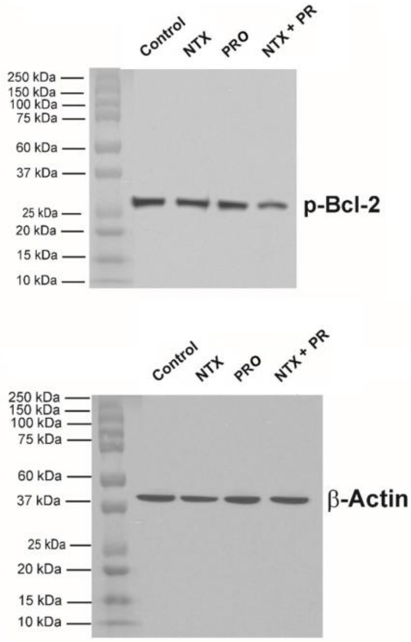


Figure 5H

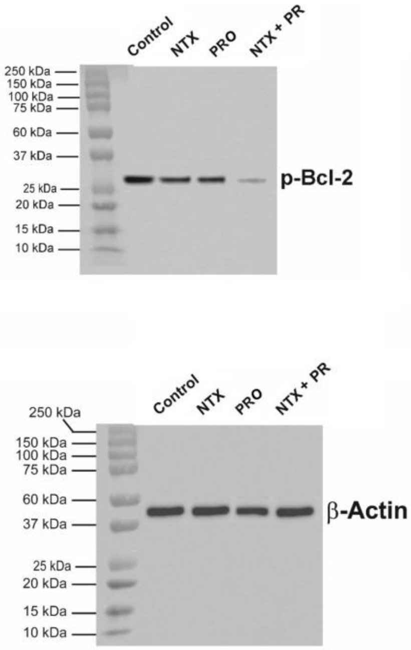


Figure 5N

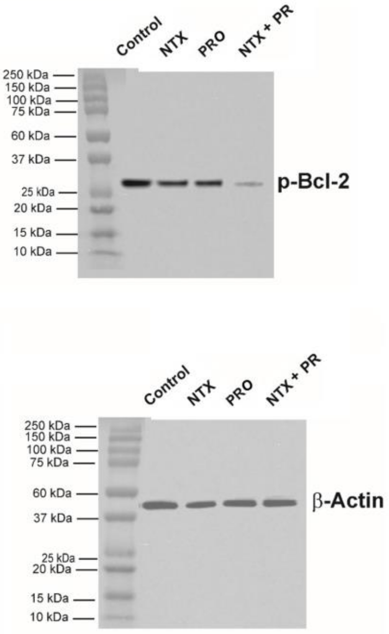

## Figure 5C

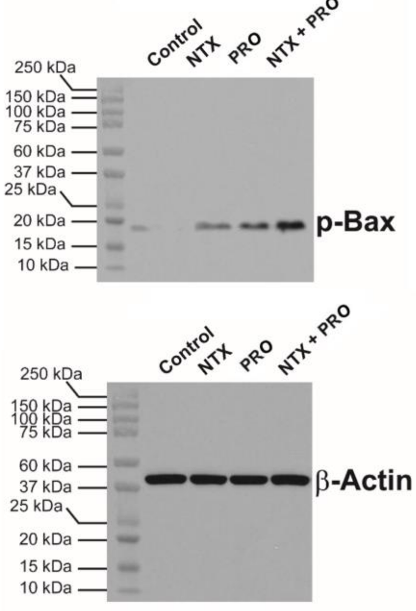


## Figure 5I

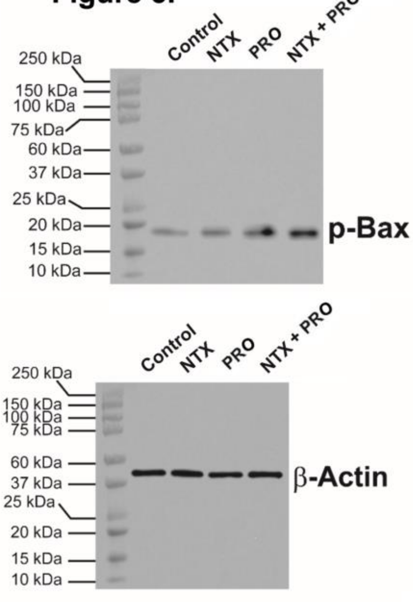


## Figure 5G

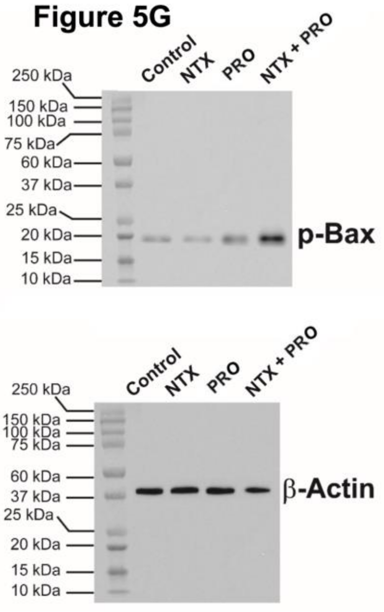


## Figure 5D

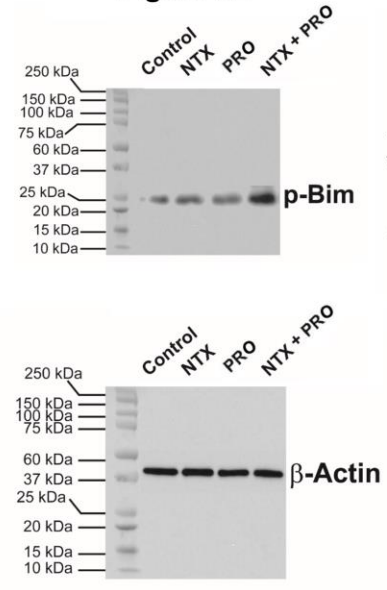


## Figure 5J

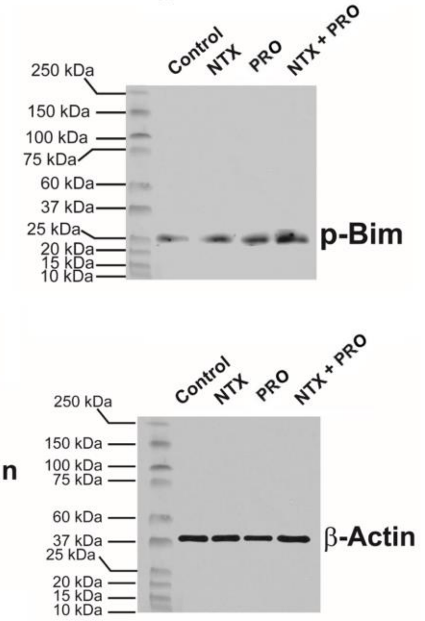


## Figure 5P

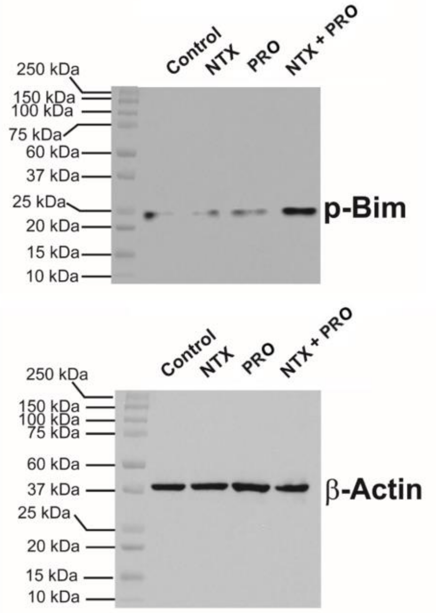

**Figure 5E**

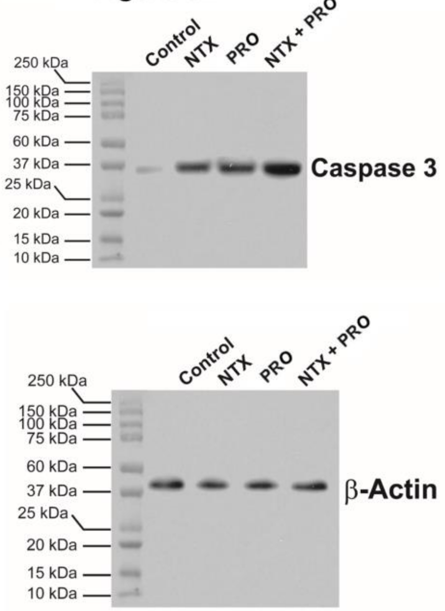


**Figure 5K**

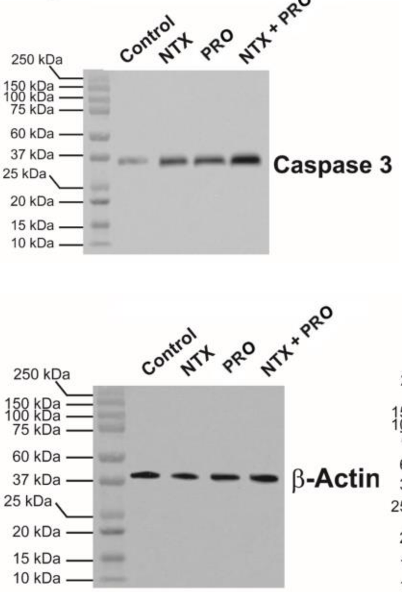


**Figure 5Q**

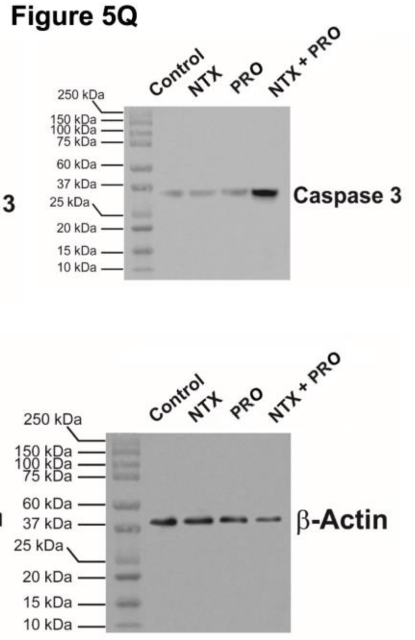


**Figure 5F**

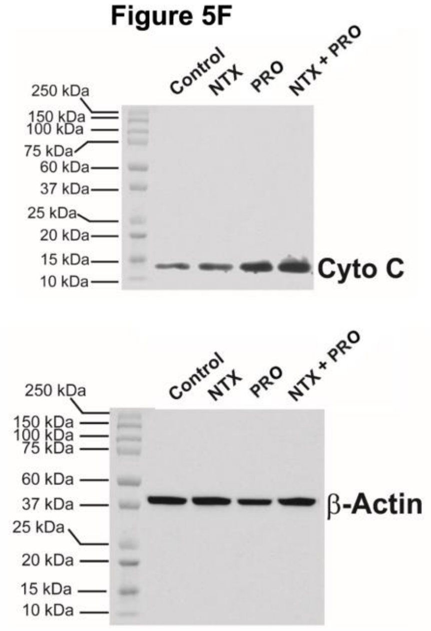


**Figure 5L**

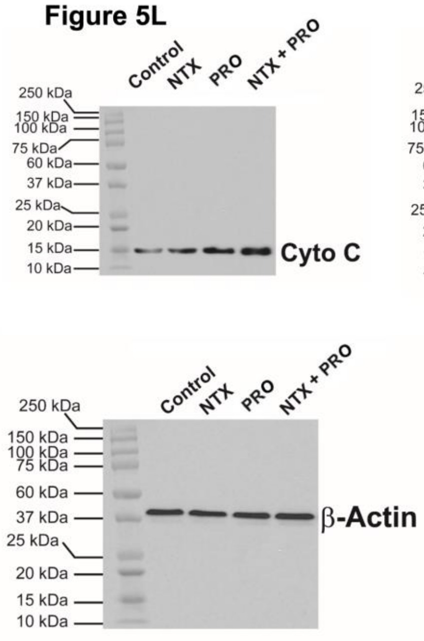


**Figure 5R**

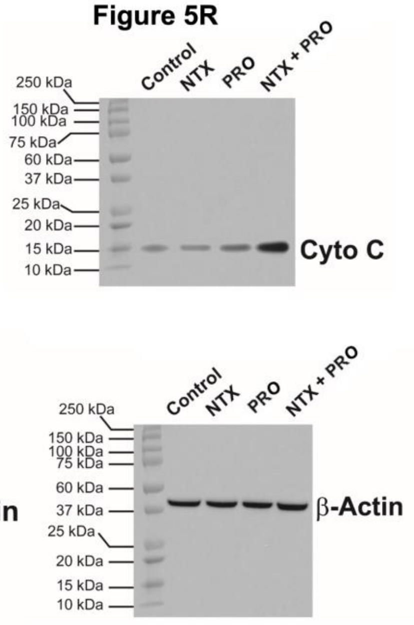

Figure 7G

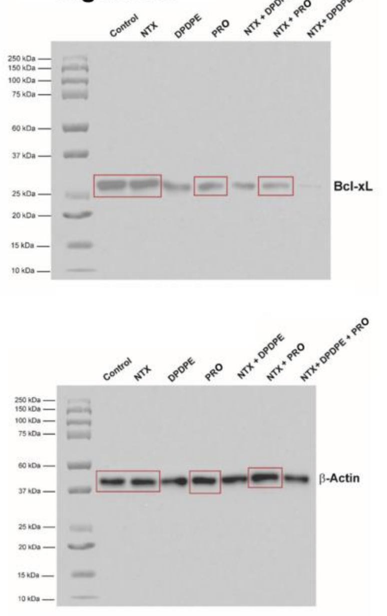


Figure 7H

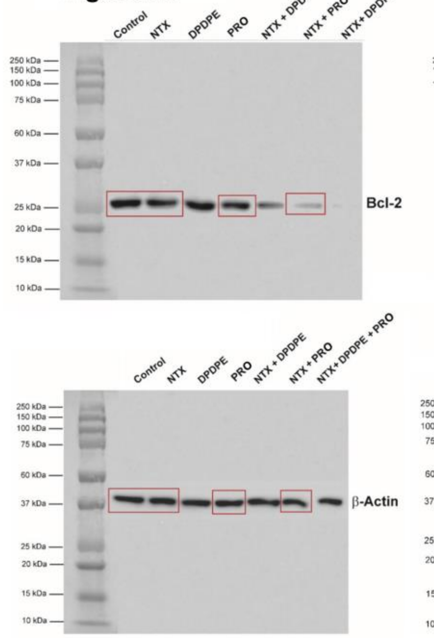


Figure 7I

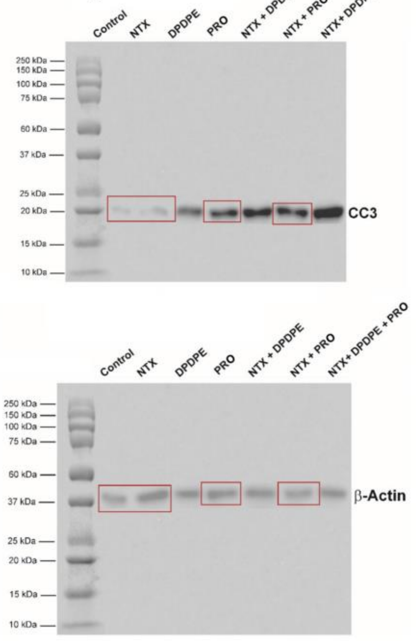


Figure 7J

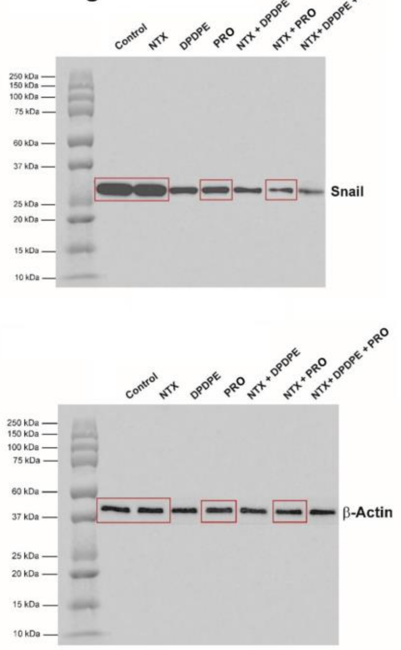


Figure 7K

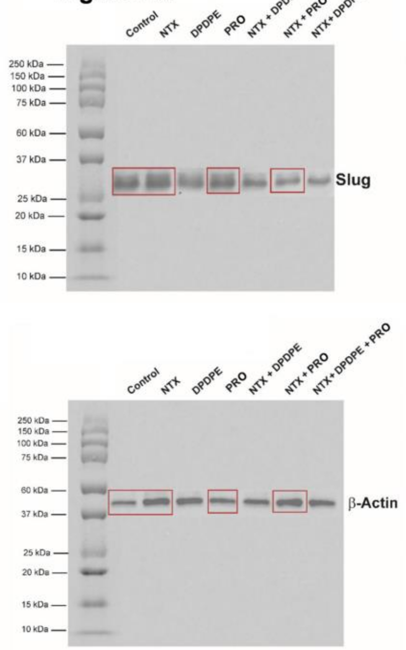


Figure 7L

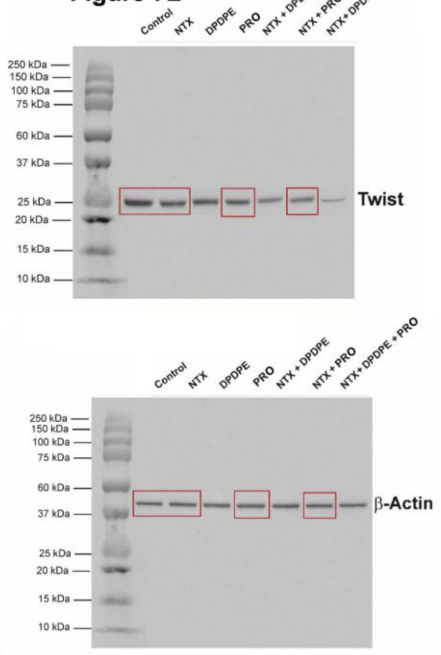

**Figure 7M**

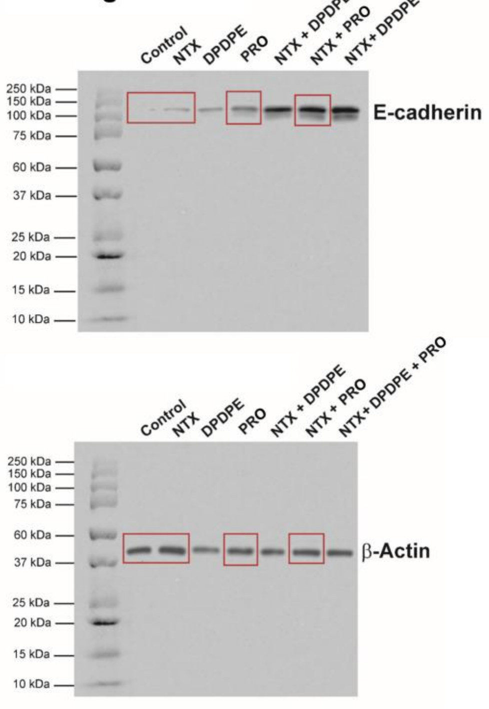


**Figure 7N**

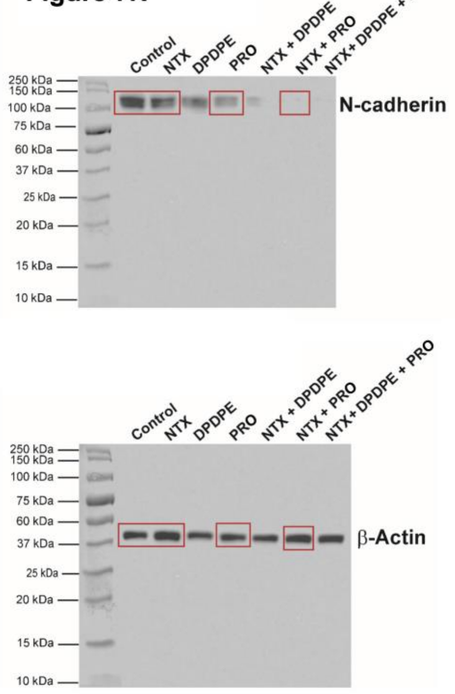


**Figure 8G**

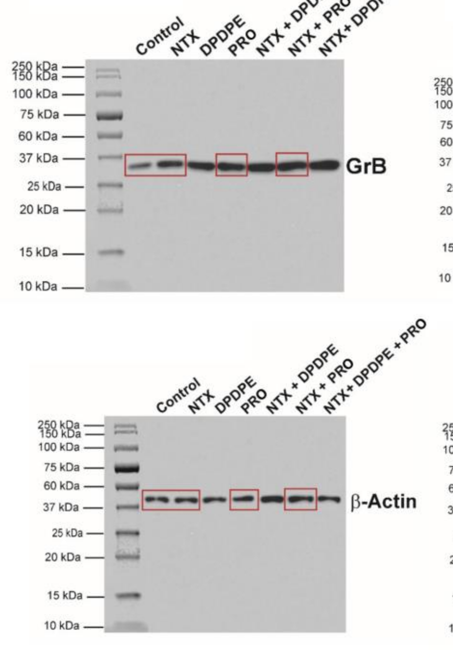


**Figure 8H**

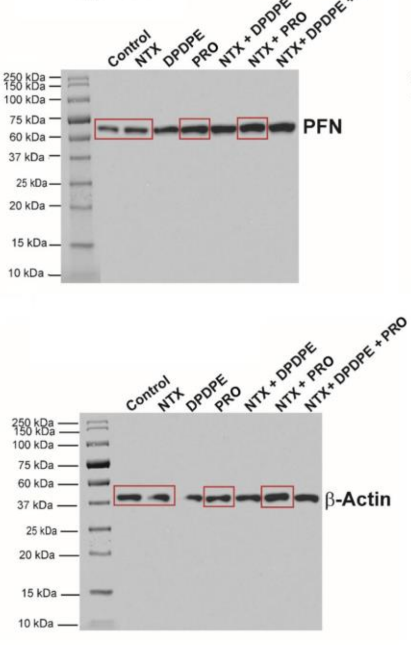


**Figure 8I**

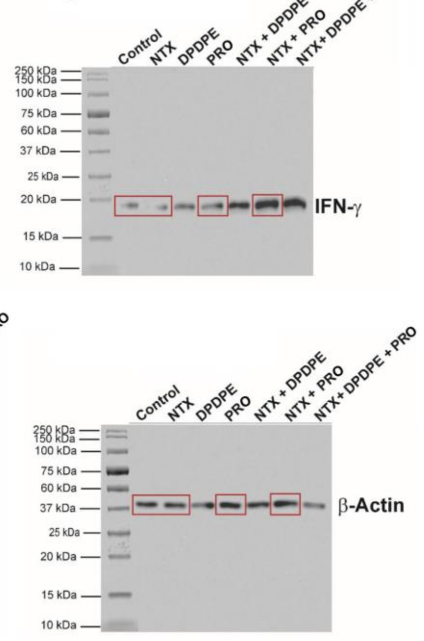

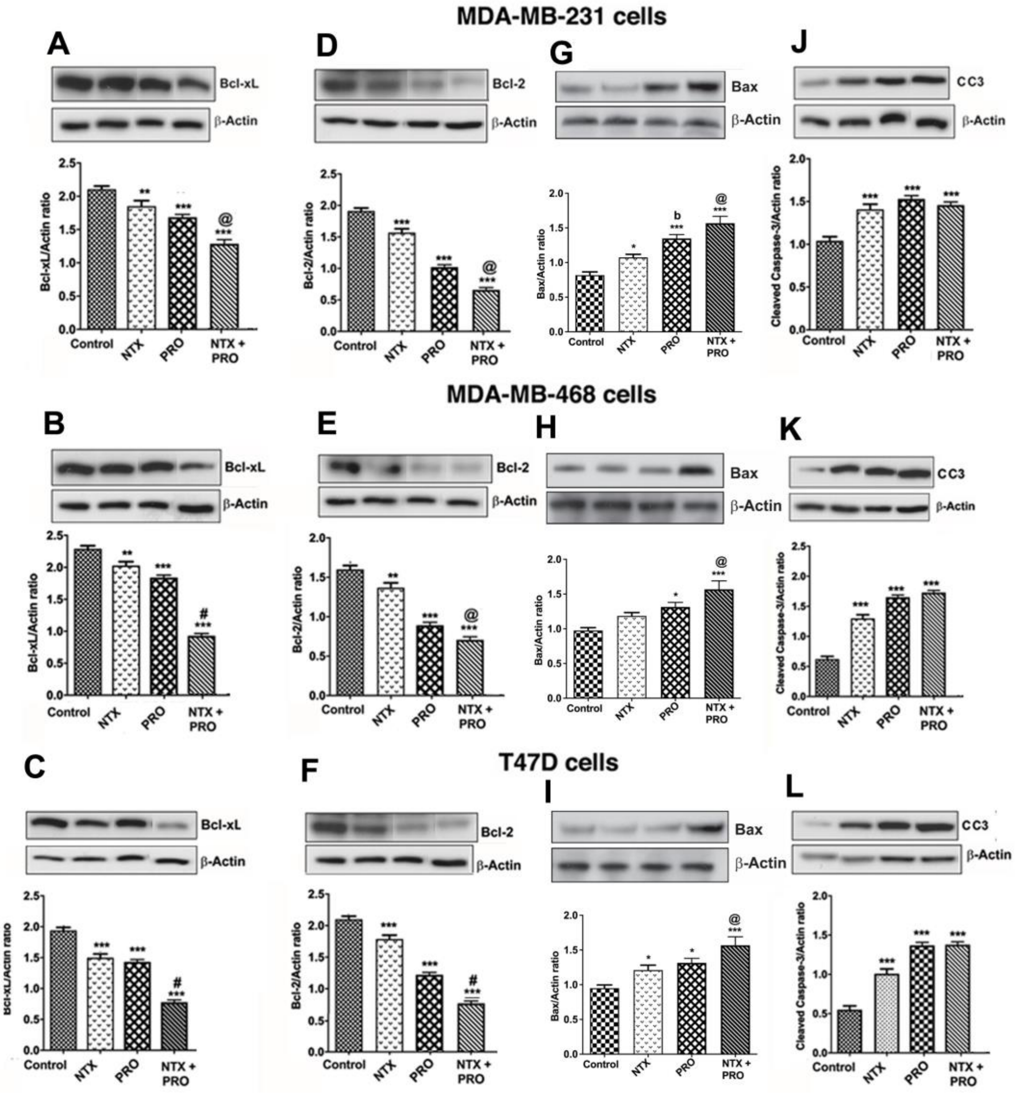


**Figure S4. Beta-adrenergic and opioidergic agents increase apoptotic protein levels in breast cancer cell lines.** Apoptotic regulatory protein levels of MDA-MB-231 (**A,D,G,J**), MDA-MB-468 (**B,E,H,K**), and T47D (**C,F,I,L**) cells following treatments with NTX, PRO, or NTX+PRO were conducted as described in materials and methods. Cells were treated with a 100 μM concentration of the beta-adrenergic and opioidergic drugs alone or in combination or vehicle alone for 48 hours. After the treatment period, some cells were extracted and used for Western blot analysis of Bcl-xL, Bcl-2, Bax, and CC3. Representative blots were presented on the top and mean densitometric values are presented as ratio of β-actin in the histograms. Data presented are mean ± SEM (*n* = 5–6 samples/group) and were analyzed using one-way ANOVA with the Newman-Keuls multiple

**Supplementary Tables**

**Table S1**: ***List of antibodies used for immunocytochemistry and Western blot analysis & cell lines used in this study***

| REAGENT or RESOURCE | Dilution | SOURCE | IDENTIFIER |
|---|---|---|---|
| Snail (C15D3) Rabbit mAb antibody | 1:250 | Cell Signaling Technology, Danvers, MA | Cat# 3879, RRID:AB_2255011 |
| Mouse Anti-SLUG Monoclonal Antibody, Unconjugated, Clone A-7 | 1:250 | Santa Cruz Biotechnology, Dallas, Texas | Cat# sc-166476, RRID:AB_2191897 |
| Twist (Twist2C1a) antibody | 1:250 | Santa Cruz Biotechnology, Dallas, Texas | Cat# sc-81417, RRID:AB_1130910 |
| Rabbit Anti-E Cadherin Polyclonal Antibody, Unconjugated | 1:1000 | Abcam, Branford, CT | Cat# ab53033, RRID:AB_868611 |
| Rabbit Anti-N Cadherin Polyclonal Antibody, Unconjugated | 1:1000 | Abcam, Branford, CT | Cat# ab18203, RRID:AB_444317 |
| Anti-Bcl-XL antibody | 1:500 | Abcam, Branford, CT | Cat# ab32370, RRID:AB_725655 |
| Anti-Bcl-2 antibody | 1:500 | Abcam, Branford, CT | Cat# ab194583, RRID:AB_2783814 |
| Cleaved Caspase-3 (Asp175) (5A1E) Rabbit mAb antibody | 1:5000 | Cell Signaling Technology, Danvers, MA | Cat# 9664, RRID:AB_2070042 |
| IFN-gamma (G-30) antibody | 1:500 | Santa Cruz Biotechnology, Dallas, Texas | Cat# sc-57208, RRID:AB_783952 |
| Granzyme B (2C5) antibody | 1:500 | Santa Cruz Biotechnology, Dallas, Texas | Cat# sc-8022, RRID:AB_2232723 |
| Perforin 1 (E-5) antibody | 1:500 | Santa Cruz Biotechnology, Dallas, Texas | Cat# sc-374346, RRID:AB_10988266 |
| Ki67 antibody - Proliferation Marker | 1:500 | Abcam, Branford, CT | Cat# ab15580, RRID:AB_443209 |
| β-Actin (8H10D10) Mouse mAb antibody | 1:5000 | Cell Signaling Technology, Danvers, MA | Cat# 3700, RRID:AB_2242334 |
| MOUSE ANTI RAT CD161 antibody | 1:500 | Bio-Rad, Philadelphia, PA | Cat# MCA1427, RRID:AB_2234351 |
| MOUSE ANTI RAT CD163 antibody | 1:500 | Bio-Rad, Philadelphia, PA | Cat# MCA342R, RRID:AB_321966 |
| Anti-μ-opioid receptor antibody | 1:1000 | Millipore, Danvers, MA | Cat# AB 5511 RRID:AB_177512 |
| Anti-μ-opioid receptor antibody | 1:1000 | Millipore, Danvers, MA | Cat# AB 1560 RRID:AB_90778 |
| Anti-β-adrenergic receptor antibody | 1:1000 | Abcam, Branford, CT | Cat# Ab61778 RRID:AB_940556 |
| Phospho-Bcl-xL (Thr47) Polyclonal Antibody | 1:500 | Thermo Fisher Scientific, Waltham, MA | Cat# PA5104974 RRID: AB_2816447 |
| Phospho-BCL-2 (Thr69) Polyclonal Antibody | 1:500 | Thermo Fisher Scientific, Waltham, MA | PA5104784 RRID: AB_2816257 |
| Caspase 3 Polyclonal Antibody | 1:500 | Thermo Fisher Scientific, Waltham, MA | Cat# PA577887 RRID: AB_2735574 |
| Bax Monoclonal Antibody (6A7) | 1:500 | Thermo Fisher Scientific, Waltham, MA | Cat# MA5-14003 RRID: AB_10979735 |
| Phospho-Bax (Ser184) Polyclonal Antibody | 1:500 | Thermo Fisher Scientific, Waltham, MA | Cat# PA5-39778 RRID: AB_2556329 |
| Phospho-Bim (Ser44, Ser104) Polyclonal Antibody | 1:500 | Thermo Fisher Scientific, Waltham, MA | Cat# PA5-105973 RRID: AB_2817372 |
| Cytochrome C Monoclonal Antibody (7H8.2C12) | 1:1000 | Thermo Fisher Scientific, Waltham, MA | Cat# 33-8500 RRID: AB_2533142 |
| CDK1 Polyclonal Antibody | 1:1000 | Thermo Fisher Scientific, Waltham, MA | Cat# PA5-82086 RRID: AB_2789247 |
| Phospho-CDK1 (Thr161) Polyclonal Antibody | 1:1000 | Thermo Fisher Scientific, Waltham, MA | Cat# PA5-105745 RRID: AB_2817144 |

**Table S2: List of antibodies & dyes used in flow cytometry**

| Cell type | *Reactivity* | *Conjugate* | *Clone* | *Company* | Catalogue # | *Dilution* |
|---|---|---|---|---|---|---|

| | | | | | | |
|---|---|---|---|---|---|---|
| Monocytes | Mouse anti rat CD11b | FITC | ED8 | Bio-Rad, Philadelphia, PA | MCA619F | 1:100 |
| B-cells | Mouse anti-rat CD45RA | APC-Cy™7 | OX-33 | BD Biosciences, San Jose, CA | 561624 | 1:100 |
| NK cells | Mouse anti-rat CD161a | PE | 10/78 | BD Biosciences, San Jose, CA | 555009 | 1:100 |
| Macrophage | Mouse anti-rat RT1B | PerCP | OX-6 | BD Biosciences, San Jose, CA | 557016 | 1:100 |
| Live/Dead cells | | DAPI | | Sigma, St. Louis, MO | D-9542 | 1μg/mL |

**Table S3: List of isotype control & beads used in flow cytometry**

| *Reactivity* | *Conjugate* | *Clone* | *Company* | Catalogue # | *Dilution* |
|---|---|---|---|---|---|
| Mouse IgG1 Negative Control | FITC | | Bio-Rad, Philadelphia, PA | MCA1209F | 1:100 |
| Mouse IgG1, κ Isotype Control | APC-Cy™7 | MOPC-21 | BD Biosciences, San Jose, CA | 557873 | 1:100 |
| Mouse IgG1, κ Isotype Control | PE | MOPC-31C | BD Biosciences, San Jose, CA | 550617 | 1:100 |
| Mouse IgG1, κ Isotype Control | PerCP | MOPC-31C | BD Biosciences, San Jose, CA | 550672 | 1:100 |
| Anti-Rat Ig, κ/Negative Control Compensation Particles Set | | | BD Biosciences, San Jose, CA | 552844 | |

**Table S4:** CI values for Individual Data Point (Concentration Values) for the combination of NTX+PRO on MDA-MB-231 cells (24 hrs)

| **Drug Concentration (uM)** | **Log (CI) Values** | **CI Values** | **Fraction Affected (Fa)** | **Effects** |
|---|---|---|---|---|
| **0.001** | **-1.362710452** | **0.04338** | **93.68** | **Synergistic** |
| **0.002** | **-1.377475138** | **0.04193** | **92.26** | **Synergistic** |
| **0.02** | **-0.658921763** | **0.21932** | **90.75** | **Synergistic** |
| **0.1** | **-0.065082606** | **0.86083** | **90.12** | **Synergistic** |
| **0.2** | **-0.312185764** | **0.48732** | **86.17** | **Synergistic** |
| **1** | **-0.050171041** | **0.8909** | **82.11** | **Synergistic** |
| 2 | 0.293976542 | 1.96778 | 82.55 | Antagonistic |
| 5 | 0.500656291 | 3.16706 | 80.53 | Antagonistic |
| 10 | 0.653350501 | 4.50143 | 78.84 | Antagonistic |
| 20 | 0.689543262 | 4.89264 | 75.55 | Antagonistic |
| 30 | 0.570937362 | 3.72338 | 71.48 | Antagonistic |
| 40 | 0.479627129 | 3.01736 | 68.23 | Antagonistic |
| 50 | 0.409635606 | 2.56824 | 65.58 | Antagonistic |
| 60 | 0.330943128 | 2.14261 | 62.97 | Antagonistic |
| 70 | 0.168939214 | 1.4755 | 59.03 | Antagonistic |
| **80** | **-0.200094546** | **0.63082** | **51.33** | **Synergistic** |
| **100** | **-0.254082433** | **0.55708** | **48.55** | **Synergistic** |
| **120** | **-0.619228157** | **0.24031** | **40.40** | **Synergistic** |
| **140** | **-0.644088949** | **0.22694** | **38.75** | **Synergistic** |
| **160** | **-1.079302866** | **0.08331** | **30.31** | **Synergistic** |

| | | | | |
|---|---|---|---|---|
| **180** | **-1.10419085** | **0.07867** | **29.09** | **Synergistic** |
| **200** | **-1.300248968** | **0.05009** | **25.39** | **Synergistic** |

**Note:** Drug concentration that have synergistic effects are highlighted in bold letters

CI values for Individual Data Point (Concentration Values) for the combination of NTX+PRO on MDA-MB-231 cells (48 hrs)

| Drug Concentration (uM) | Log (CI) Values | CI Values | Fraction Affected (Fa) | Effects |
|---|---|---|---|---|
| **0.001** | **-1.339798799** | **0.04573** | **93.6** | **Synergistic** |
| **0.002** | **-1.331055266** | **0.04666** | **92.28** | **Synergistic** |
| **0.02** | **-0.611561303** | **0.24459** | **90.78** | **Synergistic** |
| **0.1** | **-0.031073644** | **0.93095** | **90.07** | **Synergistic** |
| **0.2** | **-0.260056014** | **0.54947** | **86.26** | **Synergistic** |
| **1** | **-0.001452971** | **0.99666** | **82.2** | **Synergistic** |
| 2 | 0.347031274 | 2.22347 | 82.68 | Antagonistic |
| 5 | 0.598108446 | 3.96377 | 81.16 | Antagonistic |
| 10 | 0.614568208 | 4.11688 | 77.92 | Antagonistic |
| 20 | 0.65443935 | 4.51273 | 74.6 | Antagonistic |
| 30 | 0.54856261 | 3.53641 | 70.65 | Antagonistic |
| 40 | 0.595957828 | 3.94419 | 69.5 | Antagonistic |
| 50 | 0.372983766 | 2.36039 | 64.49 | Antagonistic |
| 60 | 0.284405916 | 1.92489 | 61.71 | Antagonistic |
| 70 | 0.025043571 | 1.05936 | 56.07 | Antagonistic |
| **80** | **-0.169180453** | **0.67736** | **51.57** | **Synergistic** |
| **100** | **-0.237629077** | **0.57859** | **48.59** | **Synergistic** |
| **120** | **-0.558210633** | **0.27656** | **41.43** | **Synergistic** |
| **140** | **-0.521318093** | **0.30108** | **40.9** | **Synergistic** |
| **160** | **-1.092534893** | **0.08081** | **30.38** | **Synergistic** |
| **180** | **-1.120388093** | **0.07579** | **29.16** | **Synergistic** |
| **200** | **-1.253599356** | **0.05577** | **26.5** | **Synergistic** |

**Note:** Drug concentration that have synergistic effects are highlighted in bold letters

CI values for Individual Data Point (Concentration Values) for the combination of NTX+PRO on MDA-MB-231 cells (72 hrs)

| Drug Concentration (uM) | Log (CI) Values | CI Values | Fraction Affected (Fa) | Effects |
|---|---|---|---|---|
| **0.001** | **-1.306888885** | **0.04933** | **92.67** | **Synergistic** |
| **0.002** | **-1.245881106** | **0.05677** | **91.53** | **Synergistic** |
| **0.02** | **-0.64535383** | **0.22628** | **89.27** | **Synergistic** |
| **0.1** | **-0.233364114** | **0.5843** | **87.33** | **Synergistic** |
| **0.2** | **-0.318369881** | **0.48043** | **84.25** | **Synergistic** |
| **1** | **-0.028603531** | **0.93626** | **80.34** | **Synergistic** |
| 2 | 0.431289767 | 2.69954 | 81.94 | Antagonistic |
| 5 | 0.577918991 | 3.78372 | 79.36 | Antagonistic |
| 10 | 0.669018259 | 4.66679 | 77 | Antagonistic |
| 20 | 0.689042339 | 4.887 | 73.55 | Antagonistic |
| 30 | 0.573506323 | 3.74547 | 69.63 | Antagonistic |
| 40 | 0.548584715 | 3.53659 | 67.49 | Antagonistic |
| 50 | 0.383129285 | 2.41618 | 63.56 | Antagonistic |
| 60 | 0.261755187 | 1.82707 | 60.42 | Antagonistic |
| 70 | 0.011308181 | 1.02638 | 55.27 | Antagonistic |
| **80** | **-0.217634888** | **0.60585** | **50.5** | **Synergistic** |
| **100** | **-0.234980019** | **0.58213** | **48.59** | **Synergistic** |
| **120** | **-0.589290235** | **0.25746** | **41.41** | **Synergistic** |
| **140** | **-0.940853281** | **0.11459** | **34.78** | **Synergistic** |
| **160** | **-1.179798541** | **0.0661** | **30.38** | **Synergistic** |
| **180** | **-1.221342368** | **0.06007** | **29.07** | **Synergistic** |
| **200** | **-1.364717362** | **0.04318** | **26.5** | **Synergistic** |

**Note:** Drug concentration that have synergistic effects are highlighted in bold letters

CI values for Individual Data Point (Concentration Values) for the combination of NTX+PRO on MDA-MB-468 cells (24 hrs)

| Drug Concentration (uM) | Log (CI) Values | CI Values | Fraction Affected (Fa) | Effects |
|---|---|---|---|---|
| **0.001** | **-1.046675304** | **0.08981** | **93.60** | **Synergistic** |
| **0.002** | **-1.034469056** | **0.09237** | **92.28** | **Synergistic** |
| **0.02** | **-0.311740398** | **0.48782** | **90.78** | **Synergistic** |
| 0.1 | 0.270089286 | 1.86247 | 90.07 | Antagonistic |
| 0.2 | 0.046791616 | 1.11376 | 86.26 | Antagonistic |
| 1 | 0.309677001 | 2.04022 | 82.20 | Antagonistic |
| 2 | 0.657722089 | 4.54697 | 82.68 | Antagonistic |
| 5 | 0.859759561 | 7.24035 | 80.61 | Antagonistic |
| 10 | 1.015967175 | 10.3745 | 78.96 | Antagonistic |
| 20 | 1.053205254 | 11.3033 | 75.69 | Antagonistic |
| 30 | 0.929193545 | 8.49559 | 71.56 | Antagonistic |
| 40 | 0.842603 | 6.9599 | 68.40 | Antagonistic |
| 50 | 0.759339923 | 5.74566 | 65.56 | Antagonistic |
| 60 | 0.690190762 | 4.89994 | 63.13 | Antagonistic |
| 70 | 0.518333604 | 3.29863 | 59.07 | Antagonistic |

| | | | | |
|---|---|---|---|---|
| 80 | 0.1532049 | 1.423 | 51.57 | Antagonistic |
| **100** | **-0.002176919** | **0.995** | **48.59** | **Synergistic** |
| **120** | **-0.293435826** | **0.50882** | **40.43** | **Synergistic** |
| **140** | **-0.199661032** | **0.63145** | **40.90** | **Synergistic** |
| **160** | **-0.774148189** | **0.16821** | **30.38** | **Synergistic** |
| **180** | **-0.802609159** | **0.15754** | **29.16** | **Synergistic** |
| **200** | **-1.008021209** | **0.09817** | **25.50** | **Synergistic** |

**Note:** Drug concentration that have synergistic effects are highlighted in bold letters

CI values for Individual Data Point (Concentration Values) for the combination of NTX+PRO on MDA-MB-468 cells (48 hrs)

| Drug Concentration (uM) | Log (CI) Values | CI Values | Fraction Affected (Fa) | Effects |
|---|---|---|---|---|
| **0.001** | **-1.054826954** | **0.08814** | **93.6** | **Synergistic** |
| **0.002** | **-1.041914151** | **0.0908** | **92.28** | **Synergistic** |
| **0.02** | **-0.318514541** | **0.48027** | **90.78** | **Synergistic** |
| 0.1 | 0.263586364 | 1.83479 | 90.07 | Antagonistic |
| 0.2 | 0.041444008 | 1.10013 | 86.26 | Antagonistic |
| 1 | 0.305198695 | 2.01929 | 82.2 | Antagonistic |
| 2 | 0.653154604 | 4.4994 | 82.68 | Antagonistic |
| 5 | 0.855554891 | 7.17059 | 80.61 | Antagonistic |
| 10 | 1.012014237 | 10.2805 | 78.96 | Antagonistic |
| 20 | 1.049683089 | 11.212 | 75.69 | Antagonistic |
| 30 | 0.926104137 | 8.43537 | 71.56 | Antagonistic |
| 40 | 0.839777111 | 6.91476 | 68.4 | Antagonistic |
| 50 | 0.756709879 | 5.71097 | 65.56 | Antagonistic |
| 60 | 0.68770104 | 4.87193 | 63.13 | Antagonistic |
| 70 | 0.516027409 | 3.28116 | 59.07 | Antagonistic |
| 80 | 0.151087789 | 1.41608 | 51.57 | Antagonistic |
| **100** | **-0.001304842** | **0.997** | **48.59** | **Synergistic** |
| **120** | **-0.295626385** | **0.50626** | **40.43** | **Synergistic** |
| **140** | **-0.201839853** | **0.62829** | **40.9** | **Synergistic** |
| **160** | **-0.776841658** | **0.16717** | **30.38** | **Synergistic** |
| **180** | **-0.805430161** | **0.15652** | **29.16** | **Synergistic** |
| **200** | **-1.011218157** | **0.09745** | **25.5** | **Synergistic** |

**Note:** Drug concentration that have synergistic effects are highlighted in bold letters

CI values for Individual Data Point (Concentration Values) for the combination of NTX+PRO on MDA-MB-468 cells (72 hrs)

| Drug Concentration (uM) | Log (CI) Values | CI Values | Fraction Affected (Fa) | Effects |
|---|---|---|---|---|
| **0.001** | **-1.051000546** | **0.08892** | **93.67** | **Synergistic** |
| **0.002** | **-1.048662481** | **0.0894** | **92.33** | **Synergistic** |
| **0.02** | **-0.331959046** | **0.46563** | **90.82** | **Synergistic** |
| 0.1 | 0.251516552 | 1.7845 | 90.13 | Antagonistic |
| 0.2 | 0.012449138 | 1.02908 | 86.25 | Antagonistic |
| 1 | 0.276758126 | 1.89129 | 82.24 | Antagonistic |

| | | | | |
|---|---|---|---|---|
| 2 | 0.617284533 | 4.14271 | 82.64 | Antagonistic |
| 5 | 0.82174928 | 6.6336 | 80.61 | Antagonistic |
| 10 | 0.97684865 | 9.48088 | 78.96 | Antagonistic |
| 20 | 1.008331848 | 10.1937 | 75.64 | Antagonistic |
| 30 | 0.884814049 | 7.67033 | 71.54 | Antagonistic |
| 40 | 0.797700234 | 6.27625 | 68.39 | Antagonistic |
| 50 | 0.715353086 | 5.19222 | 65.58 | Antagonistic |
| 60 | 0.644811666 | 4.41379 | 63.14 | Antagonistic |
| 70 | 0.471269702 | 2.95985 | 59.07 | Antagonistic |
| **80** | **0.106289512** | **0.67736** | **51.6** | **Synergistic** |
| **100** | **-0.005243055** | **0.52736** | **48.59** | **Synergistic** |
| **120** | **-0.344467804** | **0.45241** | **40.41** | **Synergistic** |
| **140** | **-0.371406744** | **0.4252** | **38.78** | **Synergistic** |
| **160** | **-0.824169396** | **0.14991** | **30.38** | **Synergistic** |
| **180** | **-0.858550227** | **0.1385** | **29.07** | **Synergistic** |
| **200** | **-1.057694247** | **0.08756** | **25.5** | **Synergistic** |

**Note:** Drug concentration that have synergistic effects are highlighted in bold letters

CI values for Individual Data Point (Concentration Values) for the combination of NTX+PRO on T47D cells (24 hrs)

| Drug Concentration (uM) | Log (CI) Values | CI Values | Fraction Affected (Fa) | Effects |
|---|---|---|---|---|
| **0.001** | **-1.362710452** | **0.04338** | **93.68** | **Synergistic** |
| **0.002** | **-1.377475138** | **0.04193** | **92.26** | **Synergistic** |
| **0.02** | **-0.658921763** | **0.21932** | **90.75** | **Synergistic** |
| **0.1** | **-0.065082606** | **0.86083** | **90.12** | **Synergistic** |
| **0.2** | **-0.312185764** | **0.48732** | **86.17** | **Synergistic** |
| **1** | **-0.050171041** | **0.8909** | **82.11** | **Synergistic** |
| 2 | 0.293976542 | 1.96778 | 82.55 | Antagonistic |
| 5 | 0.500656291 | 3.16706 | 80.53 | Antagonistic |
| 10 | 0.653350501 | 4.50143 | 78.84 | Antagonistic |
| 20 | 0.689543262 | 4.89264 | 75.55 | Antagonistic |
| 30 | 0.570937362 | 3.72338 | 71.48 | Antagonistic |
| 40 | 0.479627129 | 3.01736 | 68.23 | Antagonistic |
| 50 | 0.409635606 | 2.56824 | 65.58 | Antagonistic |
| 60 | 0.330943128 | 2.14261 | 62.97 | Antagonistic |
| 70 | 0.168939214 | 1.4755 | 59.03 | Antagonistic |
| **80** | **-0.200094546** | **0.63082** | **51.33** | **Synergistic** |
| **100** | **-0.254082433** | **0.55708** | **48.55** | **Synergistic** |
| **120** | **-0.619228157** | **0.24031** | **40.40** | **Synergistic** |
| **140** | **-0.644088949** | **0.22694** | **38.75** | **Synergistic** |
| **160** | **-1.079302866** | **0.08331** | **30.31** | **Synergistic** |
| **180** | **-1.10419085** | **0.07867** | **29.09** | **Synergistic** |
| **200** | **-1.300248968** | **0.05009** | **25.39** | **Synergistic** |

**Note:** Drug concentration that have synergistic effects are highlighted in bold letters

CI values for Individual Data Point (Concentration Values) for the combination of NTX+PRO on T47D cells (48 hrs)

| Drug Concentration (uM) | Log (CI) Values | CI Values | Fraction Affected (Fa) | Effects |
|---|---|---|---|---|
| **0.001** | **-1.339798799** | **0.04573** | **93.6** | **Synergistic** |
| **0.002** | **-1.331055266** | **0.04666** | **92.28** | **Synergistic** |
| **0.02** | **-0.611561303** | **0.24459** | **90.78** | **Synergistic** |
| **0.1** | **-0.031073644** | **0.93095** | **90.07** | **Synergistic** |
| **0.2** | **-0.260056014** | **0.54947** | **86.26** | **Synergistic** |
| **1** | **-0.001452971** | **0.99666** | **82.2** | **Synergistic** |
| 2 | 0.347031274 | 2.22347 | 82.68 | Antagonistic |
| 5 | 0.598108446 | 3.96377 | 81.16 | Antagonistic |
| 10 | 0.614568208 | 4.11688 | 77.92 | Antagonistic |
| 20 | 0.65443935 | 4.51273 | 74.6 | Antagonistic |
| 30 | 0.54856261 | 3.53641 | 70.65 | Antagonistic |
| 40 | 0.595957828 | 3.94419 | 69.5 | Antagonistic |
| 50 | 0.372983766 | 2.36039 | 64.49 | Antagonistic |
| 60 | 0.284405916 | 1.92489 | 61.71 | Antagonistic |
| 70 | 0.025043571 | 1.05936 | 56.07 | Antagonistic |

| | | | | |
|---|---|---|---|---|
| **80** | **-0.169180453** | **0.67736** | **51.57** | **Synergistic** |
| **100** | **-0.237629077** | **0.57859** | **48.59** | **Synergistic** |
| **120** | **-0.558210633** | **0.27656** | **41.43** | **Synergistic** |
| **140** | **-0.521318093** | **0.30108** | **40.9** | **Synergistic** |
| **160** | **-1.092534893** | **0.08081** | **30.38** | **Synergistic** |
| **180** | **-1.120388093** | **0.07579** | **29.16** | **Synergistic** |
| **200** | **-1.253599356** | **0.05577** | **26.5** | **Synergistic** |

**Note:** Drug concentration that have synergistic effects are highlighted in bold letters

CI values for Individual Data Point (Concentration Values) for the combination of NTX+PRO on T47D cells (72 hrs)

| Drug Concentration (uM) | Log (CI) Values | CI Values | Fraction Affected (Fa) | Effects |
|---|---|---|---|---|
| **0.001** | **-1.308918508** | **0.0491** | **92.67** | **Synergistic** |
| **0.002** | **-1.248259126** | **0.05646** | **91.53** | **Synergistic** |
| **0.02** | **-0.648474245** | **0.22466** | **89.27** | **Synergistic** |
| **0.1** | **-0.23712646** | **0.57926** | **87.33** | **Synergistic** |
| **0.2** | **-0.323096151** | **0.47523** | **84.25** | **Synergistic** |
| **1** | **-0.034520778** | **0.92359** | **80.34** | **Synergistic** |
| 2 | 0.425856887 | 2.66598 | 81.94 | Antagonistic |
| 5 | 0.571706503 | 3.72998 | 79.36 | Antagonistic |
| 10 | 1.23661031 | 17.2429 | 83 | Antagonistic |
| 20 | 0.681083777 | 4.79826 | 73.55 | Antagonistic |
| 30 | 0.564359465 | 3.66741 | 69.63 | Antagonistic |
| 40 | 0.53878103 | 3.45765 | 67.49 | Antagonistic |
| 50 | 0.372094171 | 2.35556 | 63.56 | Antagonistic |
| 60 | 0.249704535 | 1.77707 | 60.42 | Antagonistic |
| **70** | **-0.002486928** | **0.99429** | **55.27** | **Synergistic** |
| **80** | **-0.233156047** | **0.58458** | **50.5** | **Synergistic** |
| **100** | **-0.251222973** | **0.56076** | **48.59** | **Synergistic** |
| **120** | **-0.608535588** | **0.2463** | **41.41** | **Synergistic** |
| **140** | **-0.96333119** | **0.10881** | **34.78** | **Synergistic** |
| **160** | **-1.204745817** | **0.06241** | **30.38** | **Synergistic** |
| **180** | **-1.247106845** | **0.05661** | **29.07** | **Synergistic** |
| **200** | **-1.392223396** | **0.04053** | **26.5** | **Synergistic** |

**Note:** Drug concentration that have synergistic effects are highlighted in bold letters
